# A study design for modulating two components of the risk architecture of chronic pain: adverse life events and pain catastrophising

**DOI:** 10.3389/fpsyg.2026.1884214

**Published:** 2026-09-18

**Authors:** Michelle Hermes, Hannah Schmidt, Eva Beiner, Malika Renz, Stephanie Vock, Seda Karabulut, Didenur Şahin-Çevik, Andreas Meyer-Lindenberg, Sebastian Wieland, Jonas Tesarz, Heike Tost, Rolf-Detlef Treede

**Affiliations:** 1 Department of General Internal Medicine, Psychosomatics and Psychotherapy, Heidelberg University, Heidelberg, Germany; 2 Institute of Psychology, Heidelberg University, Heidelberg, Germany; 3 Department of Psychiatry and Psychotherapy, Central Institute of Mental Health (CIMH), Medical Faculty Mannheim, Heidelberg University, Mannheim, Germany; 4 Department of Psychosomatic Medicine, Johannes-Gutenberg University, Mainz, Germany; 5 Institute of General Practice and Interprofessional Care, University Hospital Tuebingen, Tuebingen, Germany; 6 Robert Bosch Center for Integrative Medicine and Health, Stuttgart, Germany; 7 German Center for Mental Health (DZPG), Heidelberg, Germany

**Keywords:** adverse life events, chronic primary pain, chronic secondary pain, eye movement desensitization and reprocessing (EMDR), pain catastrophising, prefrontal-limbic circuits, risk architecture, thalamolimbic circuits

## Abstract

**Background:**

Traumatic experiences and dysfunctional emotional responses to pain modulate pain processing via partly different neural circuits. Modulation of pain experience by brain mechanisms is partly conveyed via descending pathways through the midbrain and brainstem. We designed an intervention study using Eye Movement Desensitization and Reprocessing (EMDR) to desensitize (a) distressing memories of adverse life events (ALE), or (b) pain-related anxiety and emotion dysregulation such as pain catastrophising (PCAT). Our primary objective is to examine the extent to which EMDR efficacy on pain severity and affective symptoms is mediated via these targeted risk factors. The secondary objective is to assess the mediational effects of brain functional and structural markers and functions of the pain modulation circuitry of the brainstem.

**Methods:**

This study is a pre-post longitudinal trial with two parallel treatment arms in individuals with chronic primary or secondary pain syndromes. Primary outcomes and mediators of interest include measures of pain severity (intensity, interference, pain-related distress) and affective symptoms (anxiety, depression, tension, irritability) assessed at baseline and post-treatment. Secondary outcomes include structural and functional brain imaging (fMRI), smartphone-based ecological momentary assessment (EMA), biosampling (blood, stool), somatosensory testing (tender point count, quantitative sensory testing, pressure cuff algometry, nociceptive predictive processing) and self-reported measures on pain and affective symptoms. EMDR treatment will consist of six sessions over the course of 2 weeks. Outcome assessors will be blinded to treatment allocation. Mediational analyses will be conducted within a parallel mediation framework using structural equation modeling.

**Discussion:**

This study aims to investigate the mechanism of how an EMDR intervention with one of two different foci influences clinical outcomes by mediation analysis. We hypothesize that (1) EMDR treatment focussing on ALE will lead to a decrease in pain severity and affective symptoms through a decrease in arousal symptoms and engagement of paraventricular thalamus and ventral striatum, and (2) that EMDR treatment focussing on pain-related anxiety and emotion dysregulation will lead to a decrease in pain severity and affective symptoms through a decrease in PCAT and strengthening of prefrontal-limbic and brainstem controls.

**Clinical trial registration:**

https://www.drks.de, identifier DRKS00039123.

## 1 Background

### 1.1 Rationale and objectives of the study

The nociceptive system serves as a critical threat detection mechanism within the body. By indicating actual or potential damage to the body, the subjective sensation of pain serves as an effective warning signal that protects an individual’s physical integrity by redirecting their attention and behaviors. In the case of chronic pain syndromes, where pain persists for longer than 3 months (Treede et al., 2019; WHO, 2022), pain perception has often lost its adaptive function. Within a predictive coding framework, pain perception follows probabilistic principles (Eckert et al., 2022; Castejón et al., 2024). The brain is considered to maintain a generative model of the environment based on prior information, which is integrated with incoming sensory input. Accordingly, the central nervous interpretation of pain is not only shaped by sensory input, but also by prior experiences and beliefs. In chronic pain, pain perception may be biased toward heightened expectations of pain (Eckert et al., 2022), which might stem from psychological trauma and adverse childhood experiences (Herzog et al., 2022; Beiner et al., 2024).

Likewise, pain-related anxiety and emotion dysregulation have been identified as key factors exacerbating chronic pain (Martinez-Calderon et al., 2019; Dieu and Briganti, 2025; Renz et al., 2025). Emotion-regulatory capacities are shaped from prenatal development onward and are sensitive to environmental exposures throughout the lifespan (Haythornthwaite et al., 2024; Cervellione et al., 2025; Fan and Kang, 2025). The exposure to adverse life events (ALE), particularly during childhood, can disrupt the maturation of neurobiological systems involved in emotion regulation (Fan and Kang, 2025). Such disruptions may predispose an individual to maladaptive cognitive-affective responses to stress and pain (Haythornthwaite et al., 2024). Pain catastrophising (PCAT), characterized by rumination, magnification of pain, and a sense of helplessness, represents one such maladaptive response tendency (Martinez-Calderon et al., 2019). Studies have consistently demonstrated moderate positive associations between childhood exposure to ALEs and higher levels of PCAT (Schrepf et al., 2018; MacDonald et al., 2021). Both, trauma-focused therapeutic interventions (Tesarz et al., 2019), and treatment of pain-related health anxiety (Ashar et al., 2022) have shown promising therapeutic results in reducing pain intensity.

While both traumatic experiences and dysfunctional emotional responses to pain are thought to modulate nociceptive signal processing and pain sensations, they are hypothesized to engage different neural circuits. Adverse life events (ALE) are thought to sensitize neural pathways from the paraventricular thalamus (PVT) to the ventral striatum (VS), enhancing the emotional component of pain and promoting chronicity (Antoniou et al., 2023; Cui et al., 2024; Tang et al., 2024). Conversely, pain-related anxiety and emotion dysregulation, such as catastrophising, are proposed to weaken top-down affective regulation via prefrontal-limbic circuits, thereby exacerbating pain (Ashar et al., 2022). Modulation of pain experience by brain mechanisms is partly conveyed via descending pathways through the periaqueductal gray (PAG) and brainstem. This way, for instance placebo effect can control nociceptive signal processing at the first synapse in the spinal dorsal horn (Eippert et al., 2009).

This study aims to investigate the role of the neural circuits in pain perception by examining the differential processing of threat experiences. Specifically, we will use Eye Movement Desensitization and Reprocessing (EMDR) to desensitize distressing memories of ALEs and psychological trauma, as well as dysfunctional fears of disease and PCAT.

To elucidate the neurobiological mechanisms underlying symptom development and relief, we will investigate how targeting these different therapeutic areas affects brain function and nociceptive signal processing. We will measure changes in neural activity and connectivity within the PVT and prefrontal-limbic circuits and investigate whether these neural changes mediate the effects of EMDR on pain severity and affective symptoms. In addition, we will assess potential effects on psychophysiological measures, laboratory tests and ecological momentary assessment (EMA).

### 1.2 Study design

This study is conceptually designed as a pre-post longitudinal trial with two parallel treatment arms. The first EMDR approach will focus on targeting distressing memories related to ALEs, while the second approach will address pain-related anxiety and emotion dysregulation. These two arms serve to investigate possible mechanisms by which an EMDR approach may reduce severity of pain and affective symptoms in individuals with chronic pain. Specifically, in dimensional analyses, we will investigate to what extent treatment efficacy is related to the expression level of the corresponding risk factor (Figure 1). Participants will be allocated to one of two EMDR treatment approaches using a covariate adaptive randomization (CAR) procedure with an allocation ratio of 1:1 (Figure 2).

**FIGURE 1:**
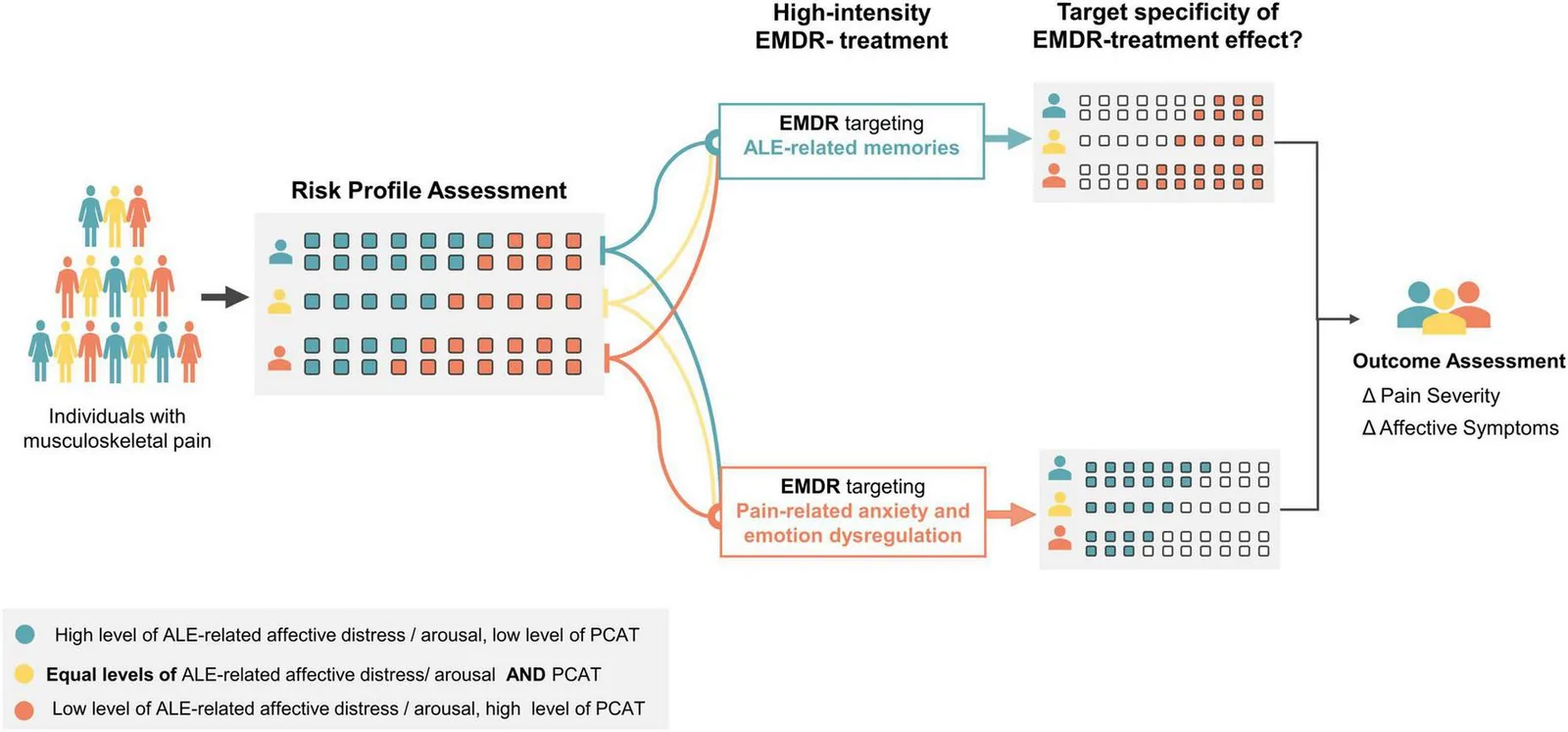
Conceptual framework illustrating the central research question of target-specific effects of Eye Movement Desensitization and Reprocessing (EMDR) in individuals with chronic pain. ALE, Adverse life events; PCAT, Pain catastrophising. Individuals’ potential risk profile is symbolically illustrated using color coding. The level of ALE-related affective distress/arousal is visualized in blue color, while the level of PCAT is visualized in orange. Yellow is used to represent individuals exhibiting equal levels ALE-related affective distress/arousal and PCAT.

**FIGURE 2:**
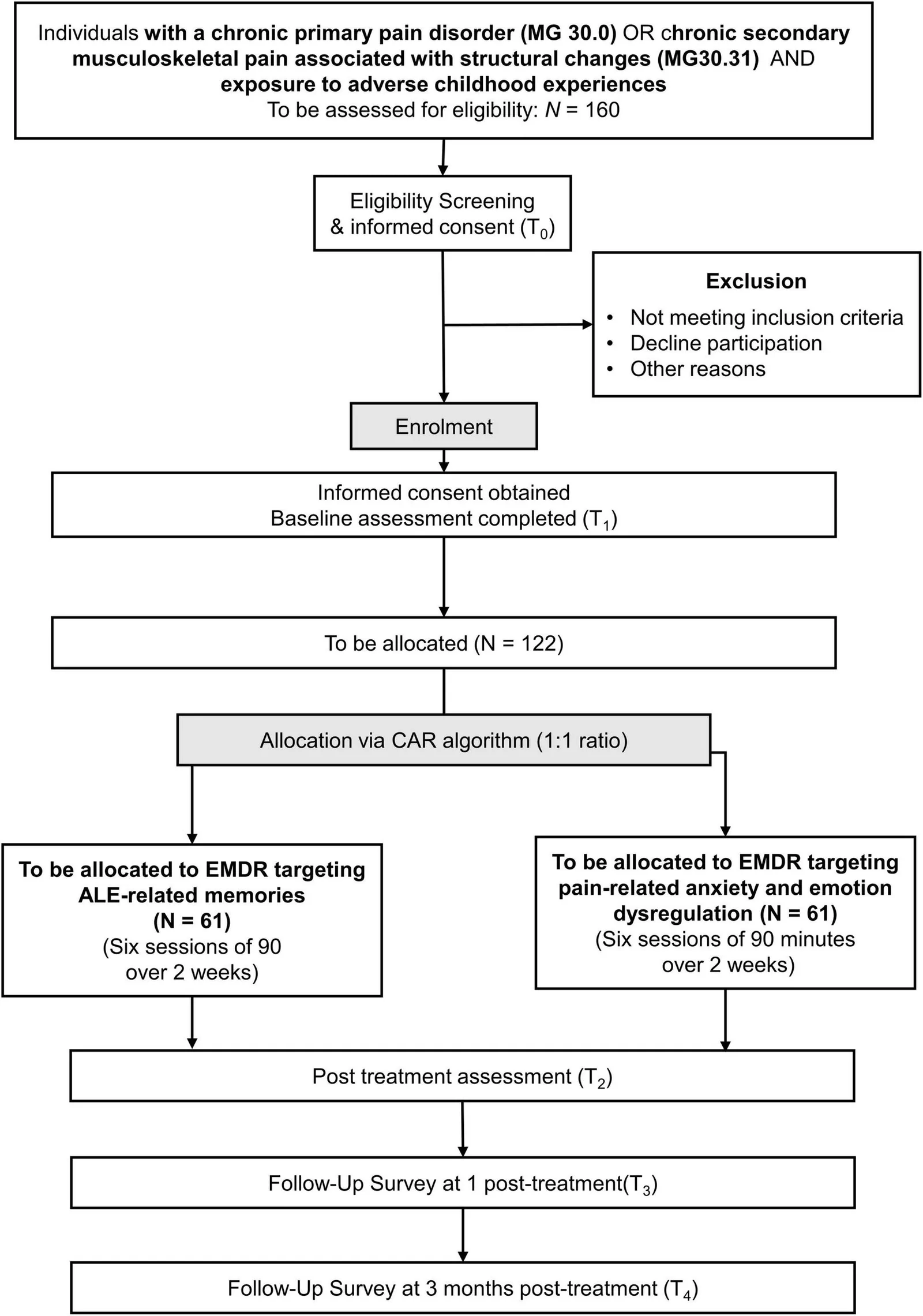
Study flowchart illustrating the intended participant flow and the designated assessment points. EMDR, Eye Movement Desensitization and Reprocessing; CAR, Covariate adaptive randomization; T0, Eligibility screening; T1, Baseline assessment; T2, Post-treatment assessment; T3, One-month follow-up; T4, three-months follow-up.

After a giving informed consent and systematic eligibility screening (T0), participants will receive a battery of psychological questionnaires via the Database REDCap and undergo baseline assessments during an in-person appointment (T1). There, the primary outcomes and mediators of interest will be examined. Additionally, (functional) magnetic resonance imaging (fMRI), quantitative sensory testing (QST), blood and stool biomarkers, and 4 weeks of EMA, will be assessed as secondary outcomes. High-intensity EMDR treatment will consist of six sessions á 90 min over the course of 2 weeks. Subsequently, all assessments that were performed at T1 will be repeated in the week after the intervention period (T2) to measure post-treatment changes. Furthermore, participants will be invited to complete follow-up surveys at 1- and 3-months post-intervention via online questionnaires. Schematic overviews of the participant timeline are provided in Table 1 and Figure 3.

**TABLE 1:** Standard protocol items: recommendations for interventional trials (SPIRIT): schedule of enrolment, interventions, and assessments.

	Trial period											
	Enrolment	Baseline	Allocation	Intervention (2–3 weeks)						Post-treatment	Follow-up	
Timepoint	T_0_	T_1_		S_1_	S_2_	S_3_	S_4_	S_5_	S_6_	T_2_	1 month (T_3_)	3 months (T_4_)
Enrolment												
Eligibility screening	X		X									
Informed consent	X											
Psychological diagnostic interview	X											
Randomization via CAR algorithm												
Intervention												
EMDR targeting ALE-related memories				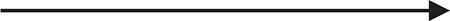								
EMDR targeting pain-related anxiety and emotion dysregulation				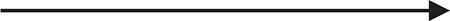								
Assessments												
Demographics	X	X								X	X	X
Mini-DIPS	X	X										
Victimization Experience Scale (VES)	X											
Impact of Event-Scale (IES-R)	X											
Depression Anxiety Stress Scale (DASS-21)										X	X	X
painDETECT		X								X	X	X
West Haven-Yale Multidimensional Pain Inventory (MPI-D)		X								X	X	X
Widespread pain index (WPI)	X	X								X	X
Symptom Severity Scale (SSS)	X	X								X	X
Perceived Stress Scale (PSS)		X								X	X	X
Pittsburgh Sleep Quality Index (PSQI)		X								X	X	X
Tampa scale of kinesiophobia (TSK-11).		X										
Fear Avoidance Beliefs questionnaire (FABQ)		X								X		
Somatosensory Testing		X								X		
Neuroimaging		X								X		
Ecological momentary assessment		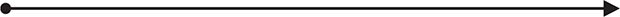							
Biosampling (blood, stool)		X								X		
Serious Adverse Events				X	X	X	X	X	X			
EMDR Measures (SUD, SUP, VoC)				X	X	X	X	X	X			
Perceived Global impression of change (PGIC)										X	X	X
Positive and Negative Experiences of psychotherapy (PNEP)										X		

S_1_-S_6_: Study intervention session 1-6. Arrows indicate continuous assessment or delivery of intervention. Template adapted from Chan et al. (2025).

**FIGURE 3:**
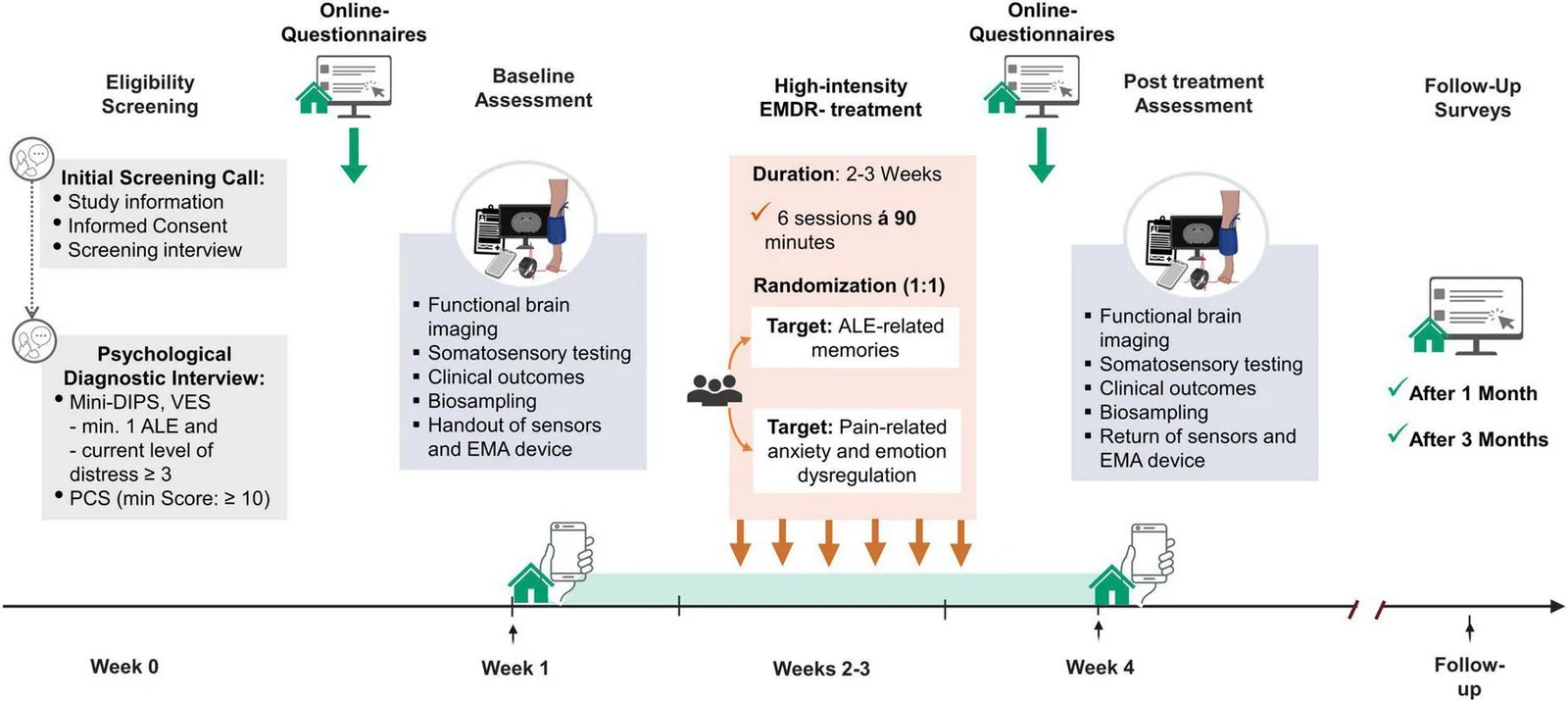
Schematic overview of the participant timeline illustrating assessment points, central assessment domains and intervention phase across the study period. In-Person outcome assessments are illustrated in blue color while the EMDR intervention is visualized in orange. Study components that can be completed by participants remotely are illustrated by a green color coding. ALE, Adverse life event; EMA, Ecological momentary assessment; EMDR, Eye Movement Desensitization and Reprocessing; PCS, Pain Catastrophising Scale; VES, Victimization Experience Schedule. Created in BioRender. Tesarz, J. (2026) https://BioRender.com/ax8wb8e.

By comparing pre- and post-treatment data, we aim to identify the mechanisms that mediate the therapeutic effects of EMDR on pain perception and emotional regulation. This design will allow us to elucidate how differentiated processing of distressing memories and pain-related catastrophising influences brain mechanisms and contributes to symptom alleviation.

In the secondary analyses we will investigate the neuronal circuits implicated in these therapeutic interventions, we will employ fMRI to assess changes in brain activity and connectivity within the PVT to VS pathway and the prefrontal-limbic circuits. Additionally, QST and EMA will be used to evaluate changes in pain perception and affective states in real time.

## 2 Methods

### 2.1 Study setting

Two German research facilities, the Medical Clinic of Heidelberg University Hospital and the Central Institute of Mental Health in Mannheim (CIMH), will be involved in this study. Neurobehavioral assessment, biomarker sampling, neuroimaging, distribution and collection of EMA devices will be performed at the CIMH in Mannheim. For both study arms, EMDR therapy will be delivered at either at Heidelberg University Hospital or the CIMH.

### 2.2 Eligibility criteria

The inclusion and exclusion criteria are chosen to cover the heterogeneity of different musculoskeletal pain conditions and diagnoses. A comprehensive overview of all inclusion and exclusion criteria is provided in Table 2.

**TABLE 2:** Overview of the in- and exclusion criteria.

Inclusion criteria	Exclusion criteria
Persisting pain for at least 3 months meeting the ICD-11 criteria of:	Current pregnancy or breastfeeding
** (a) Chronic primary pain disorder (MG 30.0):** ● Chronic Widespread Pain Syndrome including Fibromyalgia Syndrome **(MG30.01)** ● Chronic primary musculoskeletal pain (chronic primary low back pain) **(MG30.02)** ● Complex Regional Pain Syndrome **(MG30.04)**	** Inflammatory conditions:** ● Rheumatic or systemic inflammatory diseases ● Acute, pronounced inflammatory conditions accompanied by significant health impairment (e.g., fever, fatigue, chills) ○ Waiting list criterion: Assessments should only be scheduled after infection has subsided. ○ Documentation of laboratory parameters if available (exclusion/ wait list if CRP > 10 mg/L, leukocytes > 12,000/μL, ESR)
** (b) Chronic secondary musculoskeletal pain associated with structural changes (MG30.31)**	** Diseases of the CNS, e.g.:** ● Stroke ● Multiple sclerosis ● CNS-resections ● Polyneuropathy ○ Case-by-case-decision: exclusion only if symptoms are florid and overlapping with pain syndrome
** General study criteria:** ● Minimum age of 18 ● Ability to give consent ● Sufficient German language proficiency ● Willingness to participate in 4-weeks EMA	** Malignant tumors (acute)** ● Case-by-case decision for lifetime diagnosis: e.g., exclusion in case of chronic cancer-related pain (MG 30.1)
** Criteria for EMDR target availability** ● At least minimal exposure to ALEs as indicated by: (1) Identification of at least one ALE via the VES **or** via the Mini-DIPS interview (2) At least 1 ALE associated with a relevant level of ALE-related current subjective affective distress, operationalized as a current impact rating ≥ 3 **AND** ● Clinically relevant level of PCAT as indicated by a PCS total score ≥ 10 with at least one item scored ≥ 3.	** Current medication with:** ● Antipsychotic medication ● Benzodiazepines (regular intake) ● opioid therapy (defined as regular intake or use of strong opioids)
	** Psychological comorbidities and EMDR contraindications:** ● Acute psychosis ● Severe dissociative symptoms ● Fugue, severe trance states, severe neurological deficits ● Manifest mental disorders due to psychotropic substance usage ● Acute suicidal tendencies ● (indications of acute self-harm; this does not include chronic or passive suicidal ideations)
	Ongoing application process for (partial) disability pension
**Relative exclusion criteria**	**Description**
**MRI exclusion criteria according to ZIPP neuroimaging core facility**	● metal objects (denture, braces joint prostheses, metal splinters), ● active implants (pacemakers, cochlear implants, nerve stimulators, defibrillators, infusion pumps), ● previous cardiac or neurological surgical interventions, ● non-removable medicinal patches, ● piercings, ● tattoos less than 3 months old or older than 30 years, ● colored permanent makeup, ● recent occupational metal exposure for metalworkers (requiring at least 1 week of separation from metal work), ● pregnancy, ● intrauterine device placement within the past month, ● claustrophobia, ● epilepsy, ● pre-existing neurological disorders. ○Tinnitus (case-by-case-decision)
**Ongoing psychotherapy treatment**	● Case-by-case decision for waiting list ● Exclusion in case of: ○Long-term psychotherapy ○No possibility of pausing ongoing psychotherapy treatment for the entire study duration.
**Current, relevant physical therapy**	● No physical therapy in relevant pain areas (e.g., area of main pain) at least 7 days prior to in-person assessment appointments.
**Cannabis consumption**	● At least 6 weeks of abstinence prior to enrolment **AND** ● no consumption during the study
**Insufficient compliance**	● Repeatedly unavailable by phone/email ● Non-adherence to scheduled appointments (eligibility screening, assessment appointments) ● Non-compliance in online-surveys

#### 2.2.1 Key inclusion criteria

Adult outpatients (≥18 years) of both sexes are eligible to participate. Inclusion requires written informed consent, sufficient German language proficiency, and willingness to complete 4 weeks of EMA.

Additionally, participants must meet the diagnostic criteria for a chronic musculoskeletal pain condition according to ICD-11 (WHO, 2022). In line with the ICD-11 classification of chronic musculoskeletal pain we distinguish between primary and secondary pain conditions. Chronic primary musculoskeletal pain refers to pain conditions which cannot be explicitly attributed to a known disease or damage process and are associated with significant emotional distress (Nicholas et al., 2019), whereas secondary musculoskeletal pain occurs as a consequence of an identifiable underlying disease (Perrot et al., 2019). Specifically, eligible individuals will include those with either (a) a chronic primary pain disorder according to ICD-11 classification (MG 30.30; WHO, 2022), including Chronic Widespread Pain/ Fibromyalgia Syndrome (MG30.01) and/or chronic primary low back pain (MG30.02) and/or Complex Regional Pain Syndrome (MG30.04) and/or (b) chronic secondary musculoskeletal pain associated with structural changes (MG30.31).

To establish appropriate targets for EMDR therapy, participants must fulfill both of the following criteria: First, at least minimal exposure to ALEs as indicated by either (1) a score ≥ 1 in the Victimization Experience Scale (VES; Drusko et al., 2024) or (2) identification of at least one ALE via the Mini-DIPS interview (Margraf and Cwik, 2017), along with a relevant level of ALE-related current subjective affective distress operationalized as a current impact rating ≥ 3 out of 10. Second, participants must exhibit a clinically relevant level of PCAT as defined by a Pain Catastrophising Scale (PCS; Sullivan et al., 1995; Meyer et al., 2008) total score ≥ 10 with at least one item scored ≥ 3.

#### 2.2.2 Key exclusion criteria

Individuals are excluded from study participation, or from specific study components (e.g., MRI), based on medical, psychiatric, medication-related, and MRI-specific contraindications. Medical exclusions encompass acute inflammatory states, malignancies, rheumatic or systemic inflammatory diseases, diseases of the nervous system, and acute injuries. Pregnancy constitutes an absolute exclusion criterion. Psychiatric exclusions include contraindications to EMDR therapy, specifically acute psychosis, severe dissociative symptoms, severe personality disorders, and complex traumatization. Individuals receiving antipsychotic medication, benzodiazepines, or opioid therapy (defined as regular intake or use of strong opioids) are excluded. Additionally, individuals engaged in ongoing psychotherapeutic treatment at the time of enrolment are excluded unless the treatment can be paused for the study duration.

The standard MRI exclusion criteria as established by the ZIPP neuroimaging core facility will apply to all participants undergoing neuroimaging. These include the presence of metal objects (denture, braces joint prostheses, metal splinters) or active implants (pacemakers, cochlear implants, nerve stimulators, defibrillators, infusion pumps), previous cardiac or neurological surgical interventions, non-removable medicinal patches or piercings, and tattoos less than 3 months old or older than 30 years. Additional MRI-related exclusions include colored permanent makeup, recent occupational metal exposure for metalworkers (requiring at least 1 week of separation from metal work), pregnancy or intrauterine device placement within the past month, claustrophobia, epilepsy, and pre-existing neurological disorders. Tinnitus is considered a relative exclusion criterion due to possible symptom amplification following fMRI examination.

### 2.3 Interventions

EMDR therapy targets distressing memories and associated symptoms by integrating trauma-focused elements with bilateral sensory stimulation, such as left-right eye movements or bilateral tapping. It aims to reduce the emotional distress associated with specific traumatic memories or pain, thereby reducing arousal and bodily sensations. This method is based on the adaptive information processing model, which posits that unprocessed, distressing memories are a primary source of psychological suffering (Shapiro, 2001). By engaging a dual focus of attention, which is achieved by directing the patients’ focus on distressing memories while simultaneously exposing them to bilateral stimulation, EMDR promotes the reprocessing and reappraisal of these memories (Laliotis et al., 2021). For individuals with chronic pain, EMDR targets pain-related impairments by reducing fear of movement and increasing confidence in physical resilience.

EMDR treatment will be conducted at Heidelberg University Hospital or CIMH Mannheim by EMDR-trained therapists. The intervention will comprise six sessions of 90 min administered within a period of 2 weeks. This format is supported by emerging evidence that brief, intensive EMDR can be effective within a comparably short overall treatment period (De Jongh et al., 2020; Versluis et al., 2025). Following the EMDR protocol outlined by Tesarz et al. (2025), treatment begins with one session of treatment planning and preparation, followed by five further sessions of desensitization and reprocessing of either the most emotionally distressing ALE-related memories (arm 1) or pain related fears and catastrophising thoughts (arm 2). Within one session, the process of reprocessing and desensitization is repeated until the subjective level of distress or catastrophising decreases. Table 3 provides an overview of the content of the two intervention arms.

**TABLE 3:** Overview over the EMDR intervention arms.

Treatment phase	EMDR targeting ALE-related memories	EMDR targeting pain-related anxiety and emotion dysregulation
Session 1		
Treatment planning and preparation	● Establishing sustainable therapeutic relationship ● Development of a treatment plan ● Development of an “emergency protocol” with self-regulation techniques and emergency tool kit	
Psychoeducation	● neurophysiological basis of pain perception and modulation, ● role of traumatization in the etiology of chronic pain ● efficacy of EMDR in the treatment of chronic pain	● neurophysiological basis of pain perception and modulation, ● role of fear and avoidance of the pain in pain perpetuation ● efficacy of EMDR in the treatment of chronic pain
Anamnesis	● Creating an EMDR target time line of relevant adverse/ traumatic experiences ● Ranking of the most stressful experiences using the SUD scale ● Assessment of the patient’s resources.	● Identification of pain-related anxiety and emotion dysregulation based on items from PCS that score particularly high. ● Discussion of associated situations, memories, or fears about the future that are linked to these PCS items. ● Ranking of the most distressing fears using the SUD scale ● Assessment of the patient’s resources.
Absorption	● Absorption technique and EMDR to promote concentration, and relaxation.	
Session 2–5: reprocessing: performing EMDR protocol		
Possible targets	● distressing memories of ALEs ● Present day triggers related to ALEs ● ALE-related anxiety and cognitions, anticipated future situations/ triggers (flash-forward)	● pain memories associated with pain-related anxiety or emotional dysregulation (as identified in the PCS) ● Present day pain-related anxiety and cognitions ● anticipated future painful situations (flash-forward)
Session 6		
Treatment closing	● Continuation of target reprocessing in case of incomplete processing during the last session ● Reflection on the treatment process up to now ● absorption exercise, and positive treatment conclusion.	

#### 2.3.1 Therapists

Study therapists will be clinical psychologists who have completed training in EMDR-therapy. The therapists will be regularly supervised by an EMDRIA-approved consultant within the project (JT). Furthermore, they will receive external supervision every 6–8 weeks, provided by an EMDRIA-approved consultant who is not involved in the project. Therapists will administer both EMDR targeting ALE-related memories and EMDR targeting pain-related anxiety and emotion dysregulation.

#### 2.3.2 Stopping rules

In case of serious adverse events (SAEs; e.g., severe symptom deterioration, self-harm, suicidality, hospitalization, destabilization of social life), or non-compliance, the treatment may be discontinued for individual participants. Treatment must be discontinued if the participant withdraws consent. The overall study may be terminated in case of accumulating SAEs or significant changes in benefit-risk considerations. To systematically monitor for such events, SAEs will be assessed throughout the treatment period using two complementary approaches. At each EMDR session, study therapists will complete a structured SAE checklist, documenting whether any adverse events have occurred since the previous session. Additionally, potential side effects of EMDR as perceived by participants will be assessed at post treatment (T2) using a German translation of the Positive and Negative Experiences of Psychotherapy (PNEP) questionnaire (Dandachi-FitzGerald et al., 2024).

If further psychological care is needed due to acute deterioration, participants will receive post-trial care in one of the two university hospitals (University Clinic Heidelberg, Department of Psychosomatics or CIMH, Mannheim).

#### 2.3.3 Treatment fidelity

Treatment fidelity will be monitored through detailed session documentation completed by therapists after each EMDR session. This documentation includes whether EMDR was performed, the chosen EMDR target, the mode of bilateral stimulation employed, Subjective Units of Distress (SUD) at the beginning and the end of each session, Validity of Cognition (VoC) scores, and identified positive and negative cognitions. The degree of treatment fidelity will be analyzed after completion of data collection as a step of data pre-processing.

### 2.4 Outcomes

Participants will be assessed at four time points: baseline (prior to treatment allocation; T0, T1), directly after the EMDR intervention (T2), at a 1-month (T3) and 3-months follow-up (T4). At each assessment point, we will collect comprehensive data across multiple domains including questionnaire batteries on pain, stress, affective distress, and sleep quality. EMA will be conducted throughout the study period to capture momentary fluctuations in pain severity, affective symptoms, motivational drive, and stress experiences in participant’s natural environments.

Neuroimaging assessments (structural and functional MRI) will be performed at baseline and post-treatment to evaluate changes in affective and motivational functioning, as well as of interregional PVT and prefrontal-limbic connectivity. Furthermore, we perform somatosensory testing (e.g., measures of temporal summation, conditioned pain modulation and nociceptive predictive processing), and assess biomarkers of allostatic load.

#### 2.4.1 Baseline Screening and assessment of treatment target domains

All prospective participants undergo a systematic screening and a psychological diagnostic interview via phone to determine eligibility and the availability of adequate targets for the EMDR interventions.

##### 2.4.1.1 Victimization experience schedule (VES)

The German version of the VES (Drusko et al., 2024) will be used to assess lifetime exposure to adverse events. The VES is a semi-structured interview that assesses a wide range of adverse life events across the lifespan. It comprises 18 items covering several types of exposures, including interpersonal trauma and perceived discrimination experiences. Apart from the occurrence of an adverse life event, the VES captures age at exposure, frequency of exposure, duration of exposure, relationship to the perpetrator, perceived impact, and perceived helplessness (at time of exposure and at time of assessment). For each item, up to three potentially discrete events can be recorded, i.e., “exposure ≤ age 17,” “exposure > age 17,” “other events.”

Perceived impact and powerlessness related to an event, both at the time of exposure and at the time of assessment, are rated on a Likert-scale ranging from 0 “not at all” to 10 “totally.” The ratings can be aggregated into the two subscales Subjective Level of Distress (SLD) Then and SLD Now. Each subscale is computed as the mean of the respective impact and powerlessness ratings. Additionally, the response from the participants’ social circle to the respective exposure is rated by the interviewer across the two dimensions “positive response” and “negative response.” Rating is performed on a 4-point Likert-scale. For positive responses, the scale ranges from 0 “no support received” to 3 “satisfactory emotional and practical support received.” For negative responses the scale ranges from 0 “positive or neutral response received” to 3 “clear statement that the participant is to blame for or deserves what has happened.” For the items on discrimination participants are further asked to indicate the supposed reason of discrimination.

##### 2.4.1.2 Pain catastrophising

Pain-related catastrophising will be assessed using the German version of the Pain Catastrophising Scale (PCS; Sullivan et al., 1995; Meyer et al., 2008). Participants indicate their typical thoughts and feelings when experiencing pain across the three dimensions: rumination, magnification, and helplessness. The PCS comprises 19 items that are rated on a 5-point Likert scale ranging from 0 “not at all true” to 4 “always true.” The scale yields a sum score ranging from 0 to 76, with higher scores indicating greater tendencies for pain catastrophising.

#### 2.4.2 Primary outcomes

The primary outcomes of this study will be changes in clinical measures of pain severity (intensity, pain-related distress, interference; Hay et al., 2022) and affective symptoms (depression, anxiety, irritability and tension) from baseline to post-treatment.

##### 2.4.2.1 Pain intensity

To determine pain intensity, we will use the pain intensity subscale of the painDETECT questionnaire (Freynhagen et al., 2006) which is calculated as mean intensity for reported current, worst, and average pain, ranging from a 0 to 10 score.

##### 2.4.2.2 Pain-related distress

We will use the affective distress subscale from the German version of the West Haven-Yale Multidimensional Pain Inventory (MPI-D; Flor et al., 1990) to assess the emotional burden associated with chronic pain. Items are rated on a 7-point Likert scale, ranging from 0 to 6 with higher scores indicating greater pain-related distress. The scale produces a mean score.

##### 2.4.2.3 Pain interference

Pain related interference will be examined using the interference score of the MPI-D, which is computed from the average of nine items rated from 0 to 6 with higher scores indicating greater interference of pain with daily activities.

##### 2.4.2.4 Affective symptoms

We will use the German version of the Depression Anxiety Stress Scale (DASS-21; Nilges and Essau, 2021) to assess affective symptoms in participants both at baseline and follow-up. The DASS-21 contains 21 items with seven items each assessing distress on the three dimensions anxiety, depression, and stress. Items are rated on a 4-point Likert scale ranging from 0 “did not apply to me at all” to 3 “applied to me almost always.” Each scale yields a sum score with higher scores indicating greater levels of depression, anxiety or stress.

#### 2.4.3 Secondary outcomes

Secondary outcomes include spatial distribution of pain, perceived stress, sleep quality, pain related fears, clinical avoidance behaviors, somatosensory measures of nociceptive signal processing, neuroimaging outcomes, and momentary and affective symptoms in daily life. In addition, biological parameters of will be assessed.

##### 2.4.3.1 Spatial distribution of pain

To determine the spatial extent of the participants’ pain condition we will use the Widespread-Pain-Index (WPI) of the Fibromyalgia-Severity-Scale (Nilges and Essau, 2021). The WPI divides the body into 19 regions and scores the number of regions reported as painful (0–19).

Additionally, participants will be asked to indicate the characteristic distribution of their painful condition via electronic pain drawings as published before (Schmidt et al., 2025).

##### 2.4.3.2 Perceived stress

The German version of the Perceived Stress Scale (PSS; Cohen et al., 1983; Klein et al., 2016) will be used to measure participants’ level of perceived stress. The PSS comprises ten items that are rated on a 5-point Likert scale ranging from *“0 = Never*” to “*4 = Very Often*.” The PSS produces a sum score with values between 0 and 40.

##### 2.4.3.3 Sleep quality

The German version of the Pittsburgh Sleep Quality Index (PSQI; Buysse et al., 1989; Riemann and Backhaus, 1996) will be used to assess participant’s sleep quality. It comprises 19 items assessing sleep quality over the past month. A global sum score is derived from the seven sub-components “subjective sleep quality, sleep latency, sleep duration, habitual sleep efficiency, sleep disturbances, use of sleep medication, and daytime sleepiness.” The PSQI score ranges from “0” to “21” with higher values indicating poor sleep quality.

##### 2.4.3.4 Fear-pain interactions

Regarding fear pain interactions we will assess both fear of movement using the German version of the Tampa scale of kinesiophobia (TSK-11; Tkachuk and Harris, 2012; Pagels et al., 2024), and fear avoidance beliefs through the Fear Avoidance Beliefs questionnaire (FABQ; Pfingsten et al., 2000).

###### 2.4.3.4.1 Tampa scale of kinesiophobia (TSK-11)

The TSK-11 is an 11-item scale evaluating kinesiophobia across the two dimensions “somatic focus” and “activity avoidance” using a 4-point Likert scale (1 “strongly disagree” to 4 “strongly agree”). It yields a total score with values from 11 to 44, where higher values are indicative of higher kinesiophobia.

###### 2.4.3.4.2 Fear avoidance beliefs questionnaire (FABQ)

The FABQ comprises 16 items evaluating how fear-avoidance beliefs about physical activity and work may affect pain and pain-related disability. Items are scored from 0 “strongly disagree” to 6 “strongly agree.” The FABQ contains the two subscales “physical activity” and “work” with scores ranging from 0 to 24 and 0 to 42, respectively. Higher scores indicate higher levels of fear avoidance beliefs.

##### 2.4.3.5 Somatosensory testing

###### 2.4.3.5.1 Pressure cuff algometry

A computerized pressure cuff system (Nocitech, Denmark) will be used to examine perceived pain detection (PDT) and pain tolerance thresholds (PTT), spatial summation (SSP), temporal summation (TSP) and conditioned pain modulation (CPM; Graven-Nielsen et al., 2015). The cuffs will be placed on each lower leg in a position slightly below the knees, around the most voluminous portions of the gastrocnemius muscle, with participants in a reclined sitting position. Participants will be asked to quantify the perceived pain intensity during pressure application using an electronic visual analog scale (VAS), with scores ranging from “0” (no pain) to “100” (maximum pain tolerable). Further details on the pressure cuff algometry methodology can be found in Supplemental material 1.

###### 2.4.3.5.2 Nociceptive predictive processing task (NPPT)

Similar to the experimental task used in the study by Jepma et al. (2018), we will perform a nociceptive predictive processing task (NPPT) to assess the role of predictive coding in pain perception. Specifically, the NPPT aims to quantify the weighing of prior expectations against actual sensory input by manipulating the likelihood of nociceptive sensory input, i.e., by varying the likelihood of stimulus intensities (Jepma et al., 2018). Nociceptive stimuli will be induced by unilateral pressure cuff algometry in the gastrocnemius region of the dominant leg. Stimulus intensities will be calibrated to the individual pain threshold of each participant. Further information on the NPPT is provided in Supplemental material 2.

###### 2.4.3.5.3 Mechanical QST

For QST we will use an abbreviated protocol including assessment of pressure pain threshold (PPT), mechanical pain sensitivity (MPS). and dynamic mechanical allodynia (DMA).

*Pressure pain threshold:* To test sensitivity to blunt pressure in order to assess functional gain in deep tissue (Rolke et al., 2006) we will use PPT as described in the QST protocol of the German research network on Neuropathic pain (DFNS) in a uniform standard region and the region of maximal ongoing pain.

*Mechanical pain sensitivity:* MPS will be assessed according to the QST protocol by the German research network on Neuropathic pain (Rolke et al., 2006). Seven PinPrick stimulators (“The Pinprick”; MRC Systems, Heidelberg, Germany) with fixed stimulus intensities ranging from 8 to 512 mN and intensity increasing by log2 will be applied to uniform standard region and the region of maximum ongoing pain. Stimulators will be applied five times each in a balanced order and participants are instructed to rate each stimulus on a numeric rating scale (NRS) ranging from 0 “no pain” to 100 “most intense pain imaginable.”

*Dynamic mechanical allodynia:* To determine DMA a set of three light stimulators (cotton pad: force ∼ 3 Nm; cotton wool tip: force ∼ 100 Nm; brush: force ∼200–400 mN) will be applied intermingled with the PinPrick stimuli. Each of the three stimuli will be applied five times with a uniform stroking movement over the skin of approximately 1–2 cm in length. As with the PinPrick stimuli the light stimulators will be applied in a balanced order and participants are asked to rate each stimulus on a NRS (0-100). Participants will be instructed that any kind of sensation describing activation of nociceptors (“sharp,” “burning,” “aching”) is defined as “pain” and supposed to be rated with values > 0.

MPS and DMA will be calculated using the geometric mean with a constant of 0.1 added to each rating of the PinPrick or light touch stimulations (Schmidt et al., 2025).

*Manual tender point count:* We will use manual tender point (TP) count as a marker for global hypersensitivity as suggested in former ACR criteria (Wolfe et al., 1990) and in ongoing discussion about the assessment of nociplastic pain (Schmidt et al., 2025). To address methodological concerns regarding TP examination (Harth and Nielson, 2007; Wolfe and Häuser, 2011; Jensen et al., 2013), we will employ a modified examination protocol aimed at improved standardization. Specifically, we will adjust the applied digital pressure to the recommended sex-specific force thresholds (female: 476 kPa; male: 579 kPa, Pfau et al., 2014). Prior to and following TP assessment, trained assessors will perform a manual calibration of digital pressure force by applying pressure to an algometer three times. The actual applied force is recorded for each calibration attempt. With the participant in lying position, pressure is applied to each of the 18 predefined TP sites by the thumb. As described in Wolfe et al. (1990) painfulness of palpation is documented on a 5-point Likert scale (0 = no pain; 1 = mild pain: complaint of pain without grimace, flinch, or withdrawal; 2 = moderate pain: pain plus grimace or flinch; 3 = severe pain: pain plus marked flinch or withdrawal; and 4 = unbearable: participant is untouchable, withdraws without palpation). In addition to pain intensity, we will record self-reported pain-quality (i.e., numbing, radiating, shooting and/ or tingling).

###### 2.4.3.5.4 Neuroimaging

Functional and structural neuroimaging will be performed at baseline (T1) and after the EMDR intervention (T2) on a Siemens 3 Tesla MRI scanner (Siemens MAGNETOM Prisma Fit, Erlangen, Germany) using a 64-channel head coil. High-resolution structural images will be obtained with a T1-weighted 3D MPRAGE sequence (voxel size = 0.8 × 0.8 × 0.8 mm^3^, TR = 2,500 ms, TE = 3.15 ms). The fMRI session will consist of resting-state imaging and experimental task from a well-established cognitive-emotive fMRI test battery probing the processing of negative emotional and positive incentive stimuli, and working memory (face matching task, monetary incentive delay task and n-back) using a multiband echoplanar imaging (EPI) sequence (voxel size = 2.4 mm isotropic, 60 slices, TR = 800 ms, TE = 30 ms). Spin-echo EPI images with reversed phase-encoding directions (AP/PA; voxel size = 2.4 mm isotropic) will be collected for susceptibility-induced distortion correction. Diffusion-weighted images will be acquired with 1.6 mm isotropic resolution (TR = 3,350 ms, TE = 71.2 ms).

Outcome measures include regional activation and interregional connectivity estimates in PVT and prefrontal-limbic circuits, as well as brain structural measures (grey matter volume and white matter tract integrity) in regions such as the PVT, VS, PFC amygdala and anterior cingulate cortex (ACC) assessed at baseline (T1) and after EMDR intervention (T2).

###### 2.4.3.5.5 Momentary affective symptoms and pain severity in daily life

Throughout the study period, fluctuations in relevant momentary subjective experiences (e.g., perceived pain, affective instability, stress sensitivity, emotion regulation, perceived motivational drive) will be assessed with smartphone-based electronic (e-) diaries (Ebner-Priemer and Trull, 2009) and a mixed time- and location-based sampling scheme similar to those used by Renz et al. (2025). Participants will be provided with a study smartphone (Nokia 4.2) equipped with the movisensXS experience sampling software (movisens GmbH, Karlsruhe, Germany) and will receive 11 daily EMA-prompts consisting of one fixed morning-survey after waking up, one fixed questionnaire assessing sleep quality, one fixed evening survey before going to bed, and 8 randomly timed prompts throughout the day. Outcome measures include changes in these measures from baseline (T1) to post-treatment (T2).

In parallel, physical activity will be monitored continuously during the study period using LightMove4 accelerometers (sampling frequency 64 Hz). High-resolution electrocardiogram (ECG) data will be recorded for two consecutive days immediately after the fMRI session at T1 and for two consecutive days before the fMRI session at T2 using movisens MoveECG sensors (sampling frequency 1,024 Hz). Analysis of sensor data will include calculation of changes in mean non-exercise activity and indices of heart rate variability.

### 2.5 Putative mediators

To examine the mechanisms through which EMDR may influence pain and affective outcomes, we will assess changes in psychological and neurobiological variables that are hypothesized to mediate treatment effects.

#### 2.5.1 ALE-related arousal

ALE-related arousal will be operationalized as trauma-specific hyperarousal symptoms using the hyperarousal subscale of the Impact of Event Scale revisited (IES-R, German version; Maercker and Schützwohl, 1998). The hyperarousal subscale captures physiological arousal and reactivity directly related to a specific ALE. At baseline, participants will complete the IES-R in reference to the ALE which individuals rate as the most distressing (perceived impact at the time of the assessment) in the VES or the Mini-DIPS. At follow-up, the same event will be rated again, ensuring that changes in arousal reflect changes specific to the targeted memory, rather than shifts in which event is considered the most distressing. Changes in ALE-related arousal levels will be operationalized as post-treatment IES-R hyperarousal subscale scores adjusted for baseline levels within our parallel mediation framework (see Figure 4).

**FIGURE 4:**
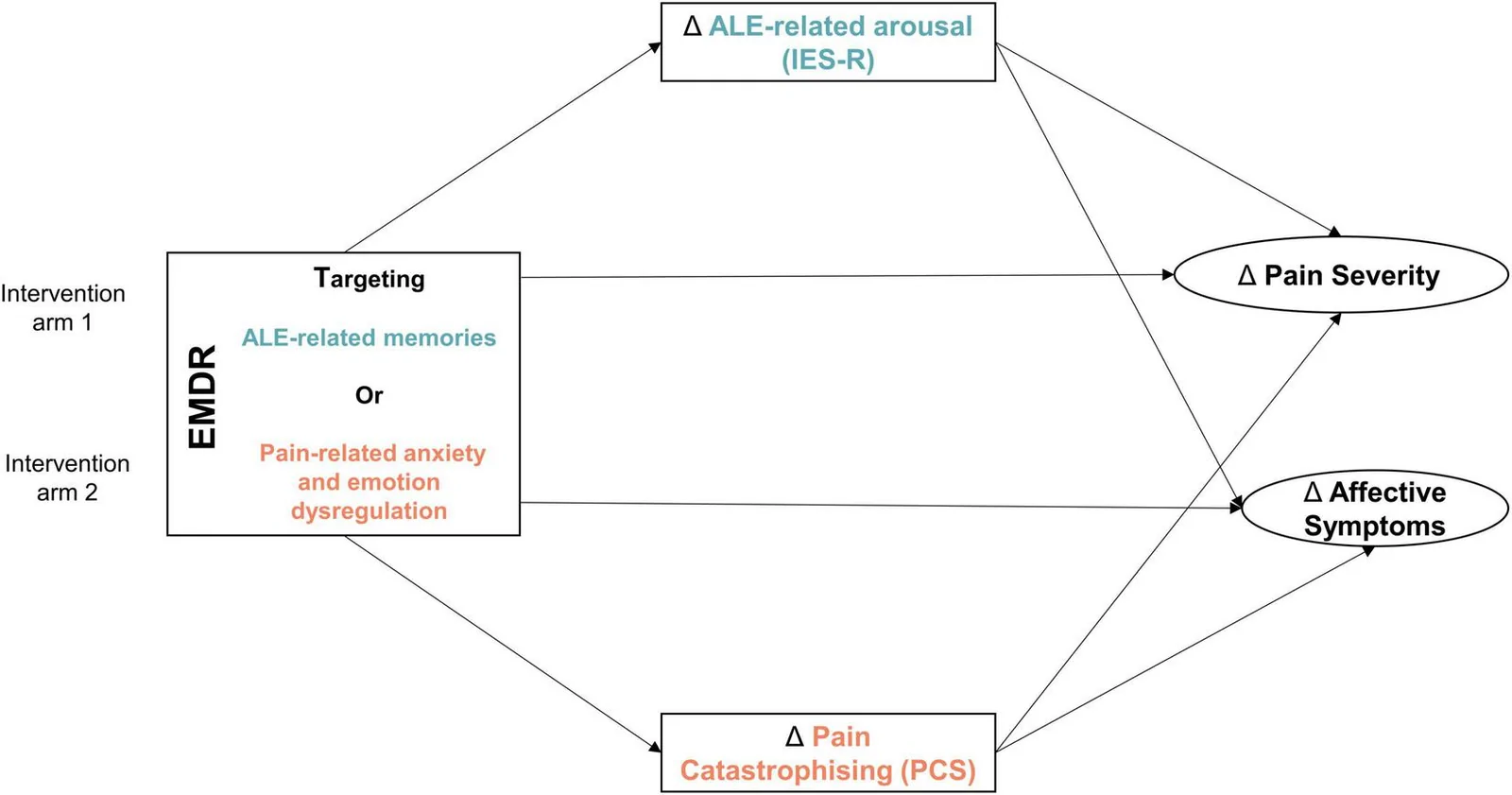
Conceptual mediation model of hypothesized mediation pathways. The model specifies treatment allocation as the independent variable, two parallel mediators: M1: changes in adverse life event (ALE) related arousal as measured by the Impact of Event Scale revisited (IES-R); M2: changes in pain catastrophising as measured by the Pain Catastrophising Scale (PCS), and two primary outcomes: (1) Changes in pain severity and (2) changes in affective symptoms. Arrows indicate hypothesized causal pathways.

#### 2.5.2 Pain catastrophising

Treatment-related changes in PCAT will serve as a key mediator in our primary analysis. Changes in PCAT will be operationalized as post-treatment PCS scores while accounting for pre-treatment levels in the mediation model (Figure 4).

#### 2.5.3 Neurobiological mediators

Changes in neuroimaging measures (as detailed in secondary outcomes above) will be examined as potential mediators of treatment effects. Specifically, we will assess whether EMDR-induced changes in: PVT and prefrontal-limbic functional connectivity, regional brain activation during emotional and motivational processing mediate improvements in pain and affective symptoms.

### 2.6 Hypotheses

The primary objective of this study is to investigate the mechanism through which an EMDR intervention influences clinical outcomes in individuals with chronic pain. We employ parallel mediation analysis to test distinct mechanistic pathways for each intervention focus (ALE, PCAT).

*H1:* ALE-focused EMDR operates through reduction in trauma-related arousal. We hypothesize that EMDR targeting ALE-related memories will lead to a significant decrease in pain severity (intensity, pain-related distress, interference) and affective symptoms (depression, anxiety, irritability and tension), mediated by a reduction in arousal symptoms from baseline to post treatment (as illustrated by the transition from blue to open squares in Figure 1).

*H2*: Pain catastrophising-focused EMDR operates through reduction in catastrophising cognitions. We hypothesize that EMDR targeting pain-related anxiety and emotion dysregulation will lead to a significant decrease in pain severity and affective symptoms mediated by improvements in affective self-regulation as reflected by reduced pain catastrophising from baseline to post-treatment (as illustrated by the transition from red to open squares in Figure 1).

#### 2.6.1 Secondary hypotheses

Of secondary interest is if both EMDR intervention foci show comparable clinical efficacy. We expect both intervention arms to produce significant and comparable improvements in pain severity and affective symptoms from baseline to post-treatment (H3).

In terms of somatosensory testing, we expect that EMDR will lead to positive effects on nociceptive processing such as decreased temporal summation, improved CPM, and reduced weighting of prior expectations over sensory information in NPPT (H4). We further anticipate that EMDR will result in gradual improvements in momentary affective function and perceived pain in daily life throughout the study period (H5).

#### 2.6.2 Neurobiological mediation hypotheses

For participants receiving EMDR targeting ALE-related memories, we anticipate changes in PVT circuitry, including increased PVT and VS response during motivational processing, as well as enhanced ACC signaling and amygdala habituation during emotion processing. Additionally, we expect changes in functional measures of interregional PVT connectivity. We hypothesize that the changes in PVT circuitry mediate the observed treatment response in pain and mood symptoms (H6).

For those undergoing EMDR targeting pain-related anxiety and emotion dysregulation we predict changes in prefrontal-limbic circuitry, i.e., decreased amygdala and increased dorsolateral prefrontal cortex and dorsal ACC signaling during emotion processing, and increased prefrontal-limbic functional connectivity during explicit emotion regulation). We expect these neural changes to mediate the observed treatment response in clinical measures of pain and affective function (H7).

#### 2.6.3 Exploratory Analyses of directionality

While H1 and H2 posit a specific direction of effects (i.e., changes in ALE-related arousal or PCAT predict changes in pain severity and affective symptoms), reversed or reciprocal associations cannot be ruled out. We will therefore draw on the high-density momentary data collected via EMA to explore this question. Specifically, the high temporal resolution of EMA data will allow for exploratory cross-lagged analyses of within-person associations between momentary affective distress, arousal, catastrophising, and pain.

### 2.7 Statistical analyses

Analyses will be performed in the intention-to-treat sample of all randomized participants. Prior to inferential analyses, all data will be subjected to extensive data preprocessing (i.e., screening for the presence and pattern of missing data, outliers, assumptions of normality, linearity, homoscedasticity, and multicollinearity). Missing data will be handled through multiple imputation.

#### 2.7.1 Descriptive statistics and outcome characterization

Descriptive statistics will be computed for sociodemographic and baseline clinical sample characteristics. Categorial variables will be presented as frequencies (*N*, %) and continuous variables as means (*SD*) and medians (*IQR*). Baseline distributions and post-treatment changes in primary outcome measures will be analyzed for the overall sample and stratified by EMDR intervention arm, sociodemographic and clinical characteristics [e.g., sex, chronic pain diagnosis (primary vs. secondary)]. Additionally, exploratory subgroup comparisons will be conducted to address potential confounders. Specifically, descriptive statistics for primary outcome measures at baseline and post-treatment will be stratified by (a) pain medication, with particular attention to gabapentinoid use (Harris et al., 2013), and (b) history of psychotherapy (Kessner et al., 2014) (no prior treatment history, recently discontinued, paused for study participation, or history predating the past 6 months).

For visualization of treatment response patterns within the two EMDR intervention arms across outcome domains, standardized effect sizes (Cohen’s *d* with 95% confidence intervals [CIs]) will be computed for all primary and secondary outcomes and visualized as forest plots. To examine whether matching of EMDR treatment targets to individual risk profiles influences treatment response patterns, outcome measures will be additionally stratified by EMDR intervention arm and allocation adequacy, operationalized by ALE-exposure level for the arm targeting ALE-related memories and baseline level of PCAT for the arm targeting pain-related anxiety and emotion dysregulation.

#### 2.7.2 Primary mediation analysis

For the primary analysis, we will test a parallel mediation model using structural equation modeling (SEM) to investigate the effect of EMDR on both primary outcomes (change in pain severity and affective symptoms, respectively) through the mediation of ALE-related arousal and level of PCAT. The model specification is illustrated in Figure 5. For inferential testing, will use bootstrap resampling (2,000 iterations) to generate 95%-CIs. Model fit will be evaluated using standard fit indices, namely model chi-square test, robust Root Mean Square Error of Approximation (RMSEA; Steiger, 1990) along with its 90%-CI, and the Standardized Root Mean Square Residual (SRMR). Values below 0.08 for the RMSEA and values below 0.10 for the SRMR are considered indicative of an acceptable fit (Browne and Cudeck, 1992; Kline, 2016).

**FIGURE 5:**
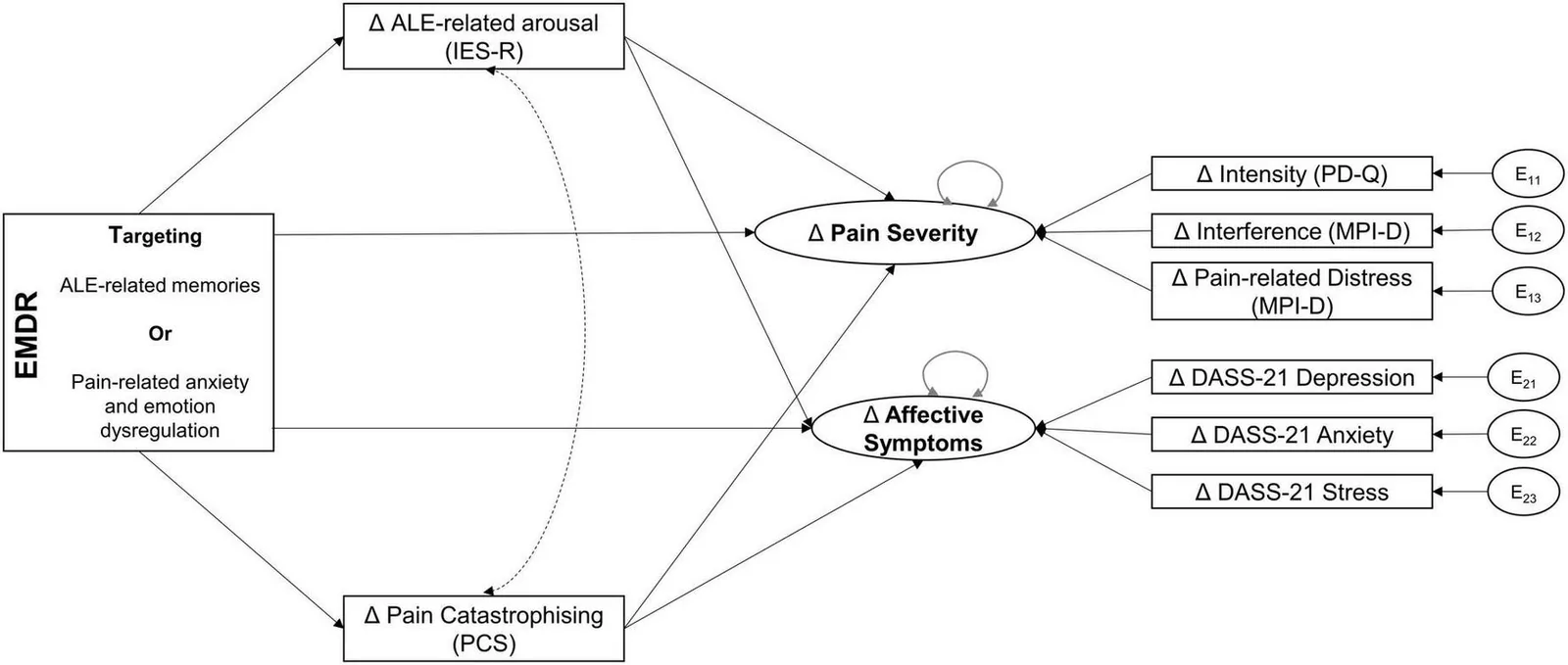
Path diagram of the parallel mediation model specification. Changes in pain severity as measured by the painDETECT questionnaire (PD-Q), in pain intensity and in pain-related distress as measured by the West Haven-Yale Multidimensional Pain Inventory (MPI-D) are specified as indicators for the latent variable “pain severity.” Changes in the latent outcome variable “affective symptoms” will be measured by the Depression, Anxiety and Stress subscale scores of the Depression Anxiety Stress Scale (DASS-21). Arrows indicate hypothesized causal pathways. Curved double-headed arrows indicate correlations between variables. Error terms (E_11_ – E_23_) represent unexplained variance. ALE, Adverse life event; IES-R, Impact of Event Scale revisited; PCS, Pain Catastrophising Scale.

### 2.8 Sample size and power calculation

A priori power analysis was performed using semPower (version 2.1.3.; Moshagen and Bader, 2024) to determine the required sample size for detecting indirect effects in our parallel mediation analysis. Assumptions on path weights were either derived from the literature or based on existing data. Based on prior systematic reviews indicating moderate to high efficacy of EMDR in treating mental disorders (Valiente-Gómez et al., 2017) and chronic pain conditions (Tesarz et al., 2014; Tesarz et al., 2019), the path coefficient from EMDR to arousal (β_11_) was set to β1 = 0.40. The path weight from EMDR to PCAT (β_21_ = 0.30) was estimated more conservatively due to paucity of evidence specifically linking EMDR and PCAT (e.g., Rosser et al., 2023). The mediator-to-outcome paths were derived from correlations observed in pre-existing data of 171 subjects with major depressive disorder and chronic pain from the SUPER study (Drusko et al., 2024; Renz et al., 2025; Schmidt et al., 2025). The observed correlations are presented in Table 4. Given that the two mediators, as well as the two outcome variables “pain severity” and “affective symptoms” are allowed to covary, we specified residual correlations of *r* = 0.30 “ALE-related arousal” and “PCAT, and of *r* = 0.20 between “pain severity” and “affective symptoms.” These values were informed by the observed correlations in the existing data.

**TABLE 4:** Observed correlations among mediator and outcome variables in the SUPER study sample which were used for power analysis (*N* = 171).

Pearson correlation	ALE-Exposure (PDS)	PCAT (PCS total)	Pain intensity (CPG)	Interference (CPG)	Pain-related distress (MPI-D)	Depression (HADS-D)	Anxiety (HADS-A)	Stress (PSS)
ALE-Exposure (PDS)	1	0.29	0.24	0.32	0.45	0.42	0.50	0.52
PCAT (PCS total)		1	.41	0.39	0.46	0.50	0.54	0.50
Pain intensity (CPG)			1	0.65	0.31	0.20	0.17	0.19
Interference (CPG)				1	0.43	0.44	0.26	0.30
Pain-related distress (MPI-D)					1	0.62	0.64	0.75
Depression (HADS-D)						1	0.65	0.64
Anxiety (HADS-A)							1	0.74
Stress (PSS)								1

Data from subsets of the SUPER study sample have been published in Drusko et al. (2024); Renz et al. (2025); Schmidt et al. (2025).

The analysis targeted a statistical power of 0.80 with a two-sided alpha level of 0.05. Results indicated that a sample size of *N* = 111 is needed to detect significant mediation effects at the desired power level. Assuming an attrition rate of 10% informed by previous studies of EMDR in chronic pain treatment (Marcus, 2008; Gerhardt et al., 2016; Maroufi et al., 2016; Ghanbari Nia et al., 2018), we aim at randomizing a total of 122 participants.

### 2.9 Recruitment

Participants will be recruited in the participating study sites (CIMH Mannheim, University Clinic Heidelberg) and cooperating doctor’s offices and clinics (e.g., pain centers at Heidelberg and Mannheim University Hospitals). Additionally, we will distribute flyers in local medical and physiotherapy offices, approach self-help groups for individuals with chronic pain, and employ online and social media advertising.

### 2.10 Assignment of interventions

After completion of the baseline assessments, participants will be allocated to one of two EMDR intervention arms with an allocation ratio of 1:1. To ensure optimal balancing of the two groups with respect to key prognostic factors we will use a CAR approach. Compared to complete randomization, CAR procedures have been shown to enhance efficiency and the power of statistical inference, and to minimize bias associated with covariate imbalances across treatment arms, thus facilitating more reliable outcomes (Ma et al., 2024).

In our study, CAR will balance treatment arms based on the following covariates: biological sex (female, male), pain diagnosis (chronic primary pain, chronic secondary pain), baseline level of PCAT as measured by the PCS, and level of ALE-exposure as measured by the SLD-then subscale of the VES. For the purpose of randomization, continuous covariate scores (PCS total score and VES SLD-then score) are categorized into three ordinal strata (mild, moderate, severe).

#### 2.10.1 Stratification thresholds

Stratification thresholds for the PCS were informed by reference data of *N* = 851 individuals (Sullivan, 1995), using percentile-based definitions (see Table 5 for the chosen percentile ranges and threshold scores).

**TABLE 5:** Stratification thresholds used for CAR for the balancing variables “baseline level of PCAT” and “level of ALE-exposure.”

PCS score (percentile-based thresholds)	Percentile ranges		Group
10–19	**25**–49		Mild
20–29	**50**–74		Moderate
30–52	**75**–100		Severe
**SLD-now mean** **(cluster-analysis-based thresholds)**	**Cluster center**	**Cluster size**	**Group**
>3	1.4	49.7%	Mild
3–5.9	4.1	31.7%	Moderate
3– 5.9	6.7	18.6%	Severe

Stratification thresholds for baseline level of PCAT have been derived from reference data on the Pain Catastrophising Scale (PCS; Sullivan, 1995). Participants are eligible for participation PCS score of ≥ 10. Bold values indicate the 25th, 50th, and 75th quartiles. Stratification thresholds for level of ALE-exposure’ were informed by pre-existing Data on the Victimization Experience (VES) Schedule subscale “Subjective level of Distress now” (SLD-now), and were determined through a two-step cluster analysis.

For the VES-derived score (SLD-now subscale), thresholds were determined using a data-driven approach. Existing VES data from participants with chronic pain from two prior studies (PerPAIN trial, *N* = 100; SUPER study, *N* = 183; total *N* = 183) were combined (Beiner et al., 2022; Schmidt et al., 2025). Given the markedly skewed distribution of SLD-then scores in this sample, a two-step cluster analysis was performed to define stratification thresholds. This approach was chosen because it appropriately handles skewed distributions and identifies natural groupings within the data, yielding more clinically meaningful and interpretable thresholds than percentile-based methods (Bennette and Vickers, 2012). Final threshold values are presented in Table 5.

#### 2.10.2 Method selection and implementation of CAR

CAR will be implemented using the R package “carat” (Ma et al., 2023), which offers several CAR methods to optimize covariate balance. To identify the most effective CAR approach for our study, we conducted a simulation-based evaluation of the methods available in carat. Based on these simulations (detailed in Supplemental material 3), Hu and Hu’s general covariate-adaptive randomization procedure (HuHuCAR; Hu and Hu, 2012) was selected, as it demonstrated superior balancing performance across multiple criteria (mean overall imbalance, within-covariate-margin imbalance).

HuHuCAR uses a biased coin approach, in which allocation probabilities are dynamically adjusted when imbalance is detected in covariate profiles and/ or group size (Hu and Hu, 2012). Specifically, the algorithm calculates a distance metric reflecting the current imbalance in these domains. In the absence of any imbalance, treatment allocation is performed with equal probability (50:50). Conversely, when imbalance is detected, allocation probabilities become unequal, with higher probability assigned to the arm that would better reduce observed imbalance. This way HuHuCAR optimizes balance in group sizes and covariate profiles while simultaneously ensuring that allocation is never deterministic, thereby protecting against selection bias.

#### 2.10.3 Randomization parameters

We chose biased coin probabilities of *p* = 0.8 and *p* = 0.2 and specified equal randomization weights of ω = 1 for overall imbalance, within stratum imbalance, and imbalance at the factor margin levels for sex, and pain diagnoses. For imbalance at the factor margin levels for PCAT and for level of ALE-exposure we specified randomization weights of ω = 2.

#### 2.10.4 Allocation concealment mechanism

Participants will be allocated sequentially as they enroll in the study, thus ensuring complete concealment of the allocation sequence and preventing investigators from predicting or influencing allocation decisions in advance. The allocation sequence will be generated by an independent study manager (DSC) who is not involved in participant assessments or delivery of EMDR treatment. Furthermore, the study manager remains blinded to the actual treatment targets, as the randomization is conducted using only the neutral labels A and B. The assignment of treatment arms to A or B was itself randomized, and only the study therapists are informed of this mapping.

##### 2.10.4.1 Blinding

Blinding of participants and therapists delivering the EMDR intervention is not possible. However, participants will not receive detailed information about what is included in the other treatment arm. Treatment allocation will be performed by the study manager (DSC), who will convey the allocation status (A or B) to the EMDR-therapists. Outcome assessors will be blinded to treatment allocation and participants will be instructed not to disclose their allocation status. Unintentional unblinding of assessors will be documented. To achieve blinded data entry, blinded assessors will enter the data into the REDCap electronic database immediately after the assessments as recommended by Juul et al. (2021). Furthermore, whenever possible, participants will complete surveys electronically using the REDCap data collection system. Finally, blinding of data pre-processing, statistical analyses, and interpretation of the results will be achieved by concealing allocation status in the REDCap database as treatment “A” and “B.” The data will be unmasked post-interpretation by the data manager (DSC).

### 2.11 Data collection and management

The study data manager will supervise data integrity and storage. To promote data quality, data collection will be performed electronically within REDCap wherever possible. All REDCap data entry fields are restricted to valid value ranges, and the majority of entry fields is designed as mandatory fields to prevent missing data. For any data that is manually entered into REDCap, a second study team member will independently verify accuracy of data entry through comparison with the original documents. At regular intervals, interim analyses will be performed to verify data integrity. Interim analyses will be performed on the entire sample without unblinding of allocation status.

#### 2.11.1 Participant retention

To promote study retention, participants will receive regular reminder emails for upcoming study appointments and online survey completion. Participants not responding to repeated survey invitations will be contacted via phone by study team members to clarify any questions and resolve potential technical issues. Those who discontinue study participation will receive a short version of the end-of-treatment survey to record the reason for discontinuation, possible side effects or SAEs related to the EMDR intervention, and to collect the primary outcomes.

#### 2.11.2 Data management

All study data will be stored pseudonymised on secure institutional servers equipped with firewall protection and backup systems. Data will be retained for a minimum of 10 years following study completion in accordance with regulatory requirements for data protection. To allow for cross-project data integration, study data will additionally be integrated into the shared data bank (PainReg) of the Heidelberg Pain Consortium (CRC1158) where feasible.

#### 2.11.3 Biological specimens

Biological samples (i.e., blood and stool) will be collected during the in-person assessments at baseline and post-treatment. At both appointments, five SSTII-tubes of serum (8.5 ml; BD 367953), three tubes of EDTA-blood (10 mL, BD 367525), and 1 PAXgene RNA tube (2.5 mL, BD 762165) will be drawn. All blood samples will be transported to the CIMH biobank within 30 min of collection for immediate processing. Following processing, samples will be stored at −80°C until further analysis. Stool samples will be collected by participants themselves no more than 1–2 days before in-person assessments using the OMNIgene Gut OM-200 kit (DNA Genotek, Ottawa, ON, Canada).

#### 2.11.4 Reporting of adverse events

Despite their potential impact on treatment outcomes, adverse events during psychotherapy are rarely assessed systematically (Vock et al., 2025). Existing instruments vary widely with regard to their conceptualization and definition of adverse events and often show unsatisfactory psychometric properties (Herzog et al., 2019). Additionally, most tools exclusively focus on negative experiences, which may introduce the risk of nocebo effects due to negative priming (Herzog et al., 2019; Moritz et al., 2019). To overcome these limitations, we will use a German translation of the recently developed PNEP questionnaire (Dandachi-FitzGerald et al., 2024). Adverse psychotherapy events will be assessed at the post-treatment survey.

The PNEP comprises two subscales measuring the occurrence, intensity and the attributed cause of both positive (33 items) and negative (36 items) psychotherapy experiences. Intensity ratings for an event that has occurred are performed on a 5-point Likert scale ranging from 0 “not at all intense” to 4 “extremely intense.” Subsequently participants are asked to indicate whether they attribute the experience entirely to the treatment, to other circumstances or a combination of both. In a final item, the perceived extent to which participants have benefited from the treatment is assessed on a 7-point Likert scale (range: −3 “strongly deteriorated,” to 3 “strongly improved”). The PNEP has been translated into German using back-and-forth translation.

## 3 Add-on projects

### 3.1 Predictive features and potential moderators

A range of sociodemographic (e.g., age, sex, gender identity), clinical (e.g., ALE exposure), behavioral, dispositional psychological (e.g., personality, trait anxiety, pain catastrophising, coping styles) and neural characteristics (e.g., baseline structural measures of PVT and prefrontal-limbic circuitry) will be considered as predictive features and potential moderators of EMDR treatment effects. Neuroimaging estimates of PVT and prefrontal-limbic circuit structure will encompass the PVT, VS, PFC, amygdala, and ACC gray matter volume, as well as the structural integrity of interconnecting white matter tracts. Additionally, potential moderators amplifying the observed brain structural and functional changes in PVT and prefrontal-limbic circuitry associated with the treatment response will be examined for each EMDR treatment modality. A detailed overview of the assessment instruments is provided in Supplemental material 4.

### 3.2 Neuro-immunological mechanisms underlying pain processing and EMDR treatment success

Previous research indicates that exposure to ALEs or chronic stress (Bussières et al., 2023; Beiner et al., 2025), emotion dysregulation (Martinez-Calderon et al., 2019; Dieu and Briganti, 2025), and neuro-immunological alterations (Findeisen et al., 2025) play an essential role in the initiation and perpetuation of chronic pain. Thus, as an add-on project, we will focus on:

(1) how ALE-exposure and pain-related anxiety may affect neuro-immunological biomarkers in individuals with chronic pain,
(2) whether these neuro-immunological signatures are related to perceived pain severity, pain processing as assessed through somatosensory testing, or other clinical pain characteristics, and
(3) exploring whether changes in neuro-immune signatures from baseline to post-treatment will predict or reflect EMDR-treatment success.

Biomaterials will include peripheral blood mononuclear cells, fluid peripheral blood components (serum and plasma), and stool samples. Outcome measures will be levels of autoreactive IgG antibodies (Goebel et al., 2021), transcriptomic profiles of mRNA and lncRNA (Mohapatra et al., 2024), and composition of gut microbiota (Guo et al., 2019).

### 3.3 Standardization and quality assessment of tender point examination

TP examination is considered a useful clinical assessment tool for distinguishing nociceptive from nociplastic pain mechanisms (Schmidt et al., 2025). However, concerns persist regarding examination burden (Harth and Nielson, 2007), variable inter-rater-reliability and the methods’ susceptibility to biases, including inconsistencies in pressure application across assessors (Wolfe and Häuser, 2011; Jensen et al., 2013) and potential sex bias due to higher pressure pain thresholds in males as compared to females (Rolke et al., 2006). To address these methodological challenges, we will conduct an add-on study investigating the quality of our refined, standardized TP examination protocol. We will recruit an independent sample of individuals with chronic pain and healthy controls who will only take part in a subset of the measures covered in the main study (initial online survey and the first in-person assessment, excluding neuroimaging, biosampling, EMDR and follow-ups). Additionally, participants from the main study who are ineligible for MRI will be included in this add-on study using their T2 assessment data. During the in-person assessment, TPs will be examined independently by three trained assessors with a recovery time of at least 10 min between examinations. Based on power analysis using the R package “kappaSize” (version 1.2; Rotondi, 2013), the target sample size was set at *N* = 83. Within this add-on project, we aim to evaluate (1) inter-rater reliability of TP examination across the independent assessors, (2) calibration accuracy and drift by comparing pre- and post-assessment force calibration measures, and (3) the relationship between calibration quality and measurement reliability. Findings from this add-on project will inform quality standards and training protocols for TP examination, potentially improving its clinical utility.
