# Supplementary material for: A study design for modulating two components of the risk architecture of chronic pain: adverse life events and pain catastrophising
Source: Front Psychol. 2026 Sep 18;17:1884214. doi: 10.3389/fpsyg.2026.1884214 (PMC13631054; doi:10.3389/fpsyg.2026.1884214)
Supplement: Supplementary file 1 [file Data_Sheet_1.pdf]

Supplemental Material

Content Overview

Supplemental Material S1: Details on Pressure Cuff Algometry 2

Supplemental Material S2: Details on the Nociceptive predictive processing task (NPPT). ... 4

Supplemental Material S3: Simulation-based evaluation of the covariate-adaptive randomisation (CAR) procedures provided carat. 6

Supplemental Material S4: Overview of the questionnaires and the assessment instruments to be employed at the different study timepoints.. 9

Supplemental References 14

Supplemental Material S1: Details on Pressure Cuff Algometry

We will use a computerised pressure cuff algometry system (Nocitech, Denmark) to assess pain detection (PDT) and pain tolerance thresholds (PTT), spatial summation (SSP), temporal summation (TSP) and conditioned pain modulation (CPM; Graven-Nielsen et al., 2015). Pressure cuffs will be positioned bilaterally over the gastrocnemius muscle while participants are seated in a reclined position. Participants will continuously rate the perceived pain intensity on an electronic visual analogue scale (VAS) ranging from ‘0’ (no pain) to ‘100’ (maximum pain tolerable). The maximum pressure will be limited to 100 kPa and participants will be able to immediately deflate the cuffs by pressing a button. During a preliminary training phase each participant will be familiarised with the cuff algometry device and trained on how to use the VAS and to stop the cuff inflation.

To record the PDT and PTT, the cuff pressure will be steadily increased at a rate of 1 kPa/s up to a maximum of 100 kPa in separate rounds on the dominant and non-dominant leg. Participants will be instructed to provide continuous VAS ratings of the perceived pressure pain intensity. Upon reaching 1cm on the VAS scale, the corresponding cuff pressure will be taken as the PDT. Once the participants’ pain tolerance is reached (as indicated by a VAS rating of 10 cm or by pressing the deflation button) the corresponding cuff pressure is used as the PTT and the cuffs will deflate automatically.

SSP refers to the increase in perceived pain intensity due to the integration of nociceptive information from multiple stimulated body sites (Graven-Nielsen et al., 2015; Nie et al., 2009). SSP will be achieved by simultaneously inflating the pressure cuffs on the dominant and non-dominant leg. The degree of SSP will be determined according to the following equations [1a, b] (Tesarz et al., 2024).

(1a) $SSP_{PDT} = \frac{PDT_{both}}{mean\ (PDT_{dominant} \cdot PDT_{non-dominant})}$ **and**

(1b) $SSP_{PDT} = \frac{PTT_{both}}{mean\ (PTT_{dominant} \cdot PTT_{non-dominant})}$.

TSP will be assessed by applying ten cuff stimuli to the dominant leg with a duration of 1s and an inter-stimulus-interval of 1s. Stimulus intensity will be set to 70% of the PTT. Participants are instructed to rate the perceived pain intensity on the VAS immediately following the first stimulus and to adjust their ratings on subsequent stimuli, if necessary, without resetting the slider to zero between stimuli. The TSP effect will be determined according to equation [2] (Tesarz et al., 2024):

(2) $$TSP = mean\ (VAS_{S8}, VAS_{S9}, VAS_{S10})\ - mean\ (VAS_{S1}, VAS_{S2}, VAS_{S3})\ .$$

We will employ both a parallel and a sequential protocol to assess CPM. The parallel CPM protocol involves applying a rapidly increasing pressure to the non-dominant leg, reaching 70% of PTT (conditioning stimulus), which is then maintained at this level. Next, a steadily increasing pressure (corresponding to the initial PTT determination) is applied to the dominant leg, serving as the test stimulus to capture the conditioned PTT. Subsequently, the sequential CPM protocol is executed. To ensure a uniform duration of the conditioning stimulus for all participants, the CS is kept constant for 110 seconds in total. After a break of 15 seconds during which no pressure is applied to the cuffs, the test stimulus is applied again to the dominant leg. Both CPM effects will be calculated as the difference between the PTT of the dominant leg during conditioning and the PTT measured at baseline.

Supplemental Material S2: Details on the Nociceptive predictive processing task (NPPT).

To examine how predictive coding contributes to pain perception we will employ nociceptive predictive processing task (NPPT). The NPPT aims to quantify how strongly individuals weigh prior expectations relative to actual sensory input by systematically varying the likelihood of different nociceptive stimulus intensities. Noxious stimuli will be delivered using unilateral pressure-cuff algometry applied to the gastrocnemius muscle of the dominant leg, with stimulus intensities calibrated to the participant's individual pain threshold. The minimum stimulus intensity will be set at a cuff pressure eliciting a score of 30 on a VAS ranging from '0' (no pain) to '100' (maximum pain tolerable). The maximum stimulus intensity will be set at a cuff pressure eliciting a VAS-score of 70. The NPPT comprises an initial learning and a subsequent test phase (Figure S1).

Learning phase. During the learning phase participants are taught the associations between visual cue stimuli and the stimulus intensity to be expected. Participants are presented with a visual cue (semicircles with edges to the left/right or up/down) and are asked to indicate the anticipated pain intensity on the VAS. Subsequently, a speedometer image representing the associated stimulus intensity is shown as visual feedback. The learning phase consists of two blocks with 20 trials each.

Test phase. In the test phase, participants are again presented with the visual cue stimuli and asked to rate the anticipated pain intensity on the VAS. Instead of visual feedback, cuff pressure will be administered. After this pressure stimulus, participants will again provide an intensity rating on the VAS. The test phase consists of four blocks with 20 trials each.

Data analysis. NPPT data will be analysed using a modified version of the winning computational model described by Jepma et al. (2018), following the pre-registered analysis plan of Reichert et al. (2024).

Figure S 1

Illustration of the learning and test phases of the NPPT.

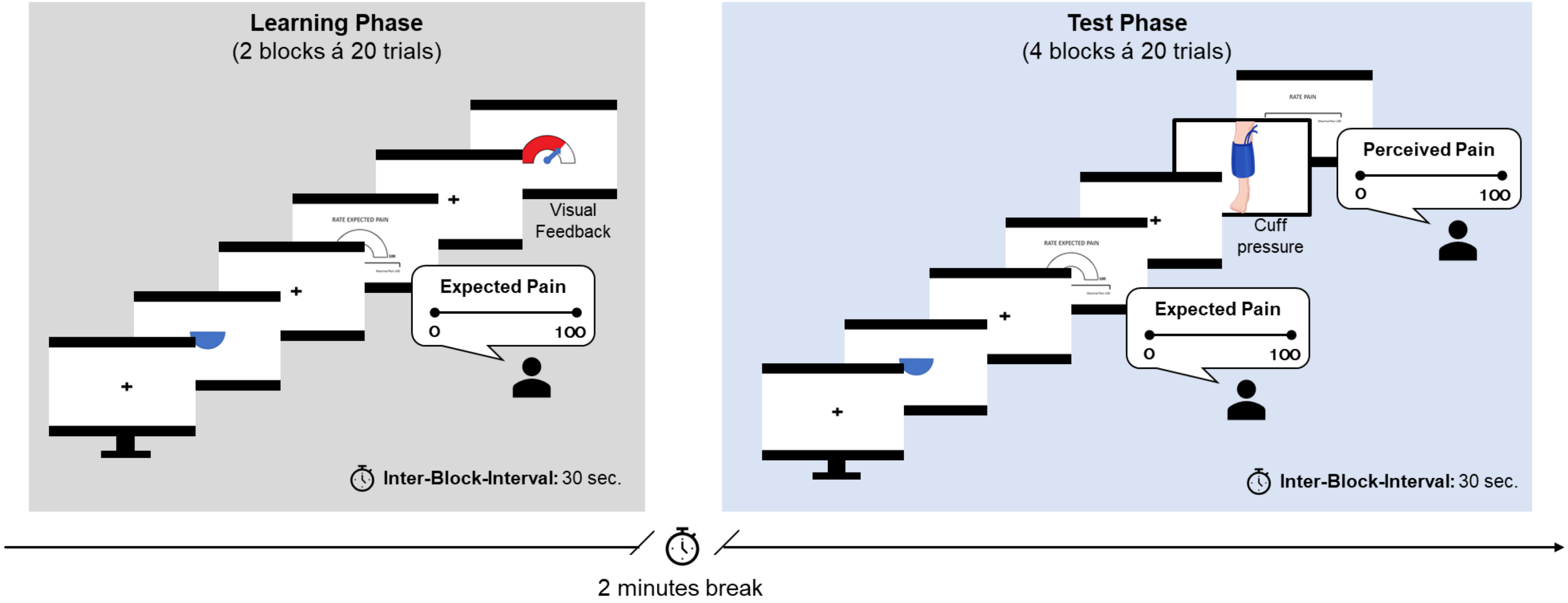

Supplemental Material S3: Simulation-based evaluation of the covariate-adaptive randomisation (CAR) procedures provided carat.

To identify the most suitable CAR approach available in the R package 'carat', we simulated the expected covariate-profiles for our sample and compared the balancing performance of each approach. Expectations regarding covariate distribution were informed from prior study data (PerPAIN trial, *N* = 100; SUPER study, *N* = 83; total N = 183; (Beiner et al., 2022; Schmidt et al., 2025). The following CAR approaches were compared with regard to balancing performance as measured by absolute overall imbalance, within-stratum imbalance and absolute within-covariate-margin-imbalance: Hu and Hu's randomisation (HuHuCAR), Pocock and Simon's minimisation (PocSimMIN), stratified biased coin design (StrBCD), tratified permuted block randomisation (StrPBR) covariate-adjusted biased coin design (AdjBCD), Atkinson's DA-optimal biased coin design (DoptBCD). Table S1 shows the simulation parameters used. As illustrated in Figure S2, HuHuCAR tended to showed best overall balancing performance and was therefore chosen for CAR in our study.

Table S1

Parameters used for simulation of balancing performance of the CAR methods available in carat.

Parameter	Value
No. participants per simulation	100
No. iterations	500
No. covariates	4
**Assumed probabilities for covariate levels (*pr)*:**	
Biological sex (male/ female)	0.5
Pain diagnosis	0.5
Baseline level of PCAT (mild/ moderate,/ severe)	..33
Level of ALE-exposure	3
- Mild	0.5
- Moderate	0.3
- Severe	0.2
**Randomisation weights (ω):**	
Overall imbalance,	1
Within-stratum imbalance	1
Within-covariate-margin imbalance (sex)	1
Within-covariate-margin imbalance (pain diagnosis)	1
Within-covariate-margin imbalance (PCAT)	1
Within-covariate-margin imbalance (ALE-exposure)	1
Biased coin probability (*p*)	.8
Block size for stratified randomisation	4
Degree of randomness parameter for AdjBCD (a > 0)	3

Note. The biased coin probability (*p*) controls the degree to which the algorithm favours assignment to the arm leading to best balancing results. The parameter $a > 0$ controls the degree of randomness of assignment for AdjBCD. Allocation gets more deterministic as $a \rightarrow \infty$.

Figure S 2

Comparison of Balancing performance with regard to overall imbalance, within-stratum imbalance, and within-covariate-margin imbalance (sex).

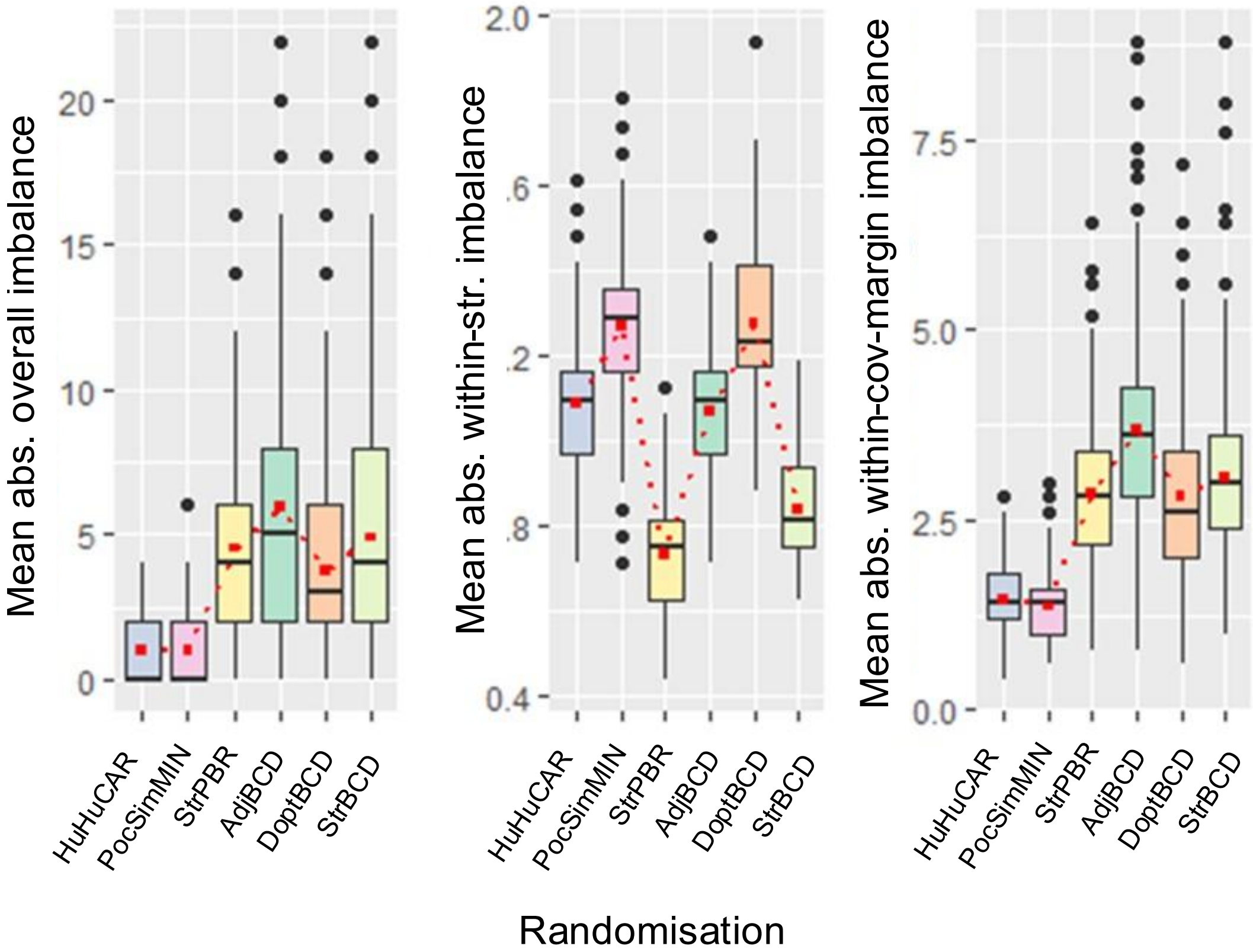

Supplemental Material S4: Overview of the questionnaires and the assessment instruments to be employed at the different study timepoints

Purpose	Instrument	Screening Calls	Baseline Survey	Baseline Assessment	Intervention	Post-Treatment Survey	Post-Treatment Assessment	1-Month Follow-Up	3- Month Follow-Up
**Eligibility Screening**	Screening questions on demographic data. language skills, sex, pain anamnesis, somatic and mental health	x							
	Screening for medication	x							
	Screening for MRI exclusion criteria	x							
Sample characteristics	Sociodemographic Data		x						
	Urbanicity		x						
	Social Network Index (SNI)		x						
	MacArthur Scale of Subjective Social Status		x						
Health & Functioning	General health (e.g. physical acticvity, smoking, alcohol, treatment efforts)		x						
	Somatic Symptom Disorder (SSD-12)		x			x			
	WHO Disability Assessment Schedule 2.0 (WHODAS 2.0)		x						
**Sleep quality**	Pitsburg Sleep Quality Index (PSQI)		x			x		x	x
Quality of life	Quality of life SF (SF-12)		x			x		x	x
	WHO Disability Quality of Life (WHOQOL)		x						
Pain characteristics	Structured Pain Interview		x	x			x		
	Digital Pain Drawings			x			x		

Purpose	Instrument	Screening Calls	Baseline Survey	Baseline Assessment	Intervention	Post-Treatment Survey	Post-Treatment Assessment	1-Month Follow-Up	3- Month Follow-Up
	West Haven-Yale Multidimensional Pain Inventory (MPI-D; Flor et al., 1990)		x			x		x	x
	Pain Experience Scale (Geissner, 1995)		x			x			
	Pain-Related Self-Statements and Contol Scale (FSK; Flor et al., 1993)		x			x			
	Chronic Pain Grade (von Korff et al., 1990)		x						x
	painDETECT (Freynhagen et al. 2006)		x			x			
	Widespread pain index (WPI)	x				x		x	x
	Symptom Severity Scale (SSS)	x				x		x	x
	Somatic Symptom Scale 8 (SSS-8)		x			x		x	x
	Pain Sensitivity Questionnaire (PSQ)		x			x			
	Douleur Neuropathique 4 (DN4)			x			x		
**Somatosensory Testing**	Mechanical QST (PPT, MPS, DMA)			x			x		
	Tender Point Assessment			x			x		
	Pressure Cuff Algometry			x			x		
	Nociceptive Predictive Processing Task			x			x		
**Fear-Pain-Interactions**	Fear Avoidance Beliefs Questionnaire (FABQ) (Pfingsten et al., 2000)		x			x			
	Whiteley Index (WI-d; Glöckner-Rist et al., 2014)		x			x		x	x
	Pain Catastrophizing Scale (PCS)	x				x		x	x

Purpose	Instrument	Screening Calls	Baseline Survey	Baseline Assessment	Intervention	Post-Treatment Survey	Post-Treatment Assessment	1-Month Follow-Up	3- Month Follow-Up
	Tampa Scale of Kinesiophobia (TSK-11)		x						
	Fear of Pain Questionnaire (FPQ, McNeil et al., 1998)		x						
**Trauma history**	Childhood Trauma Questionnaire (CTQ, Bernstein et al., 2003)		x						
	Posttraumtic Diagnostic Scale (PDS)		x						
	Vicitimization Experience Scale (VES)	x							
	Impact of Event-Scale (IES-R; Maercker et al 1998)	x				x		x	x
	Life Event Questionnaire		x						
**Stress**	Perceived Stress Scale (Cohen et al., 1983)		x			x		x	x
	Trier Inventory for Chronic Stress (Schulz & Schlotz, 1999)		x						
**Emotional Distress & psychological comorbidities**	Mini-DIPS	x							
	Hospital Anxiety and Depression Scale (Herrmann et al., 1995)		x			x		x	x
	Depression Anxiety Stress Scale (DASS-21)		x			x		x	x
	State-Trait Anxiety Inventory (Trait)		x						
	Brief Symptom Inventory (Franke, 2000)		x			x			
	Beck-Depressions-Inventory		x						
	Positive and Negative Affect Schedule (PANAS)		x			x		x	x

Purpose	Instrument	Screening Calls	Baseline Survey	Baseline Assessment	Intervention	Post-Treatment Survey	Post-Treatment Assessment	1-Month Follow-Up	3- Month Follow-Up
Additional aspects	Relationship Questionnaire (RQ2)		x						
	Cognitive Emotion Regulation Questionnaire (CERQ)		x			x		x	x
	Self-Report Measure for the Assessment of Emotion Regulation Skills (SEK-27)		x			x		x	x
	NEO Five-Factor Inventory (NEO-FFI)		x						
	Toronto Alexithymia Scale (TAS-20)		x						
	Resilience Scale (RS-13)		x						
	Mindful Attention and Awareness Scale (MAAS)					x		x	x
**Interventional effects**	Serious Adverse Events [documented by therapist]				x				
	EMDR Measures (SUD, SUP, VoC) [documented by therapist]				x				
	Perceived Global impression of change (PGIC)					x		x	x
	Positive and Negative Experiences of psychotherapy (PNEP)					x			
	Patient Questionnaire on Therapy Expectation and Evaluation (PATHEV)				x	x			
	Medication Changes					x		x	x

Purpose	Instrument	Screening Calls	Baseline Survey	Baseline Assessment	Intervention	Post-Treatment Survey	Post-Treatment Assessment	1-Month Follow-Up	3- Month Follow-Up
**Neuroimaging**	Structural Imaging		x				x		
	Field Maps		x				x		
	Resting State		x				x		
	N-Back Task		x				x		
	Face Matching Task,		x				x		
	Monetary Incentive Delay Task		x				x		
	Diffusion-Weighted Imaging		x				x		
**Biosampling**	Stool (OMNIgene Gut Kit)		x				x		
	Bood:		x				x		
	- SSTII-Serum		x				x		
	- EDTA		x				x		
	- PAXgene RNA		x				x		

Supplemental References

Beiner, E., Baumeister, D., Buhai, D., Löffler, M., Löffler, A., Schick, A., Ader, L., Eich, W., Sirazitdinov, A., Malone, C., Hopp, M., Ruckes, C., Hesser, J., Reininghaus, U., Flor, H., & Tesarz, J. (2022). The PerPAIN trial: a pilot randomized controlled trial of personalized treatment allocation for chronic musculoskeletal pain-a protocol. *Pilot Feasibility Stud*, *8*(1), 251. https://doi.org/10.1186/s40814-022-01199-6

Graven-Nielsen, T., Vaegter, H. B., Finocchietti, S., Handberg, G., & Arendt-Nielsen, L. (2015). Assessment of musculoskeletal pain sensitivity and temporal summation by cuff pressure algometry: a reliability study. *Pain*, *156*(11).

Jepma, M., Koban, L., van Doorn, J., Jones, M., & Wager, T. D. (2018). Behavioural and neural evidence for self-reinforcing expectancy effects on pain. *Nat Hum Behav*, *2*(11), 838–855. https://doi.org/10.1038/s41562-018-0455-8

Nie, H., Graven-Nielsen, T., & Arendt-Nielsen, L. (2009). Spatial and temporal summation of pain evoked by mechanical pressure stimulation. *European Journal of Pain*, *13*(6), 592–599. https://doi.org/10.1016/j.ejpain.2008.07.013

Reichert, J., Ehmsen, J. F., Sophie, H. Y., Larenz, J., & Tesarz, J. (2024). The influence of expectations on pain processing: An experimental approach with computational modeling. https://doi.org/10.17605/OSF.IO/7VDSW

Schmidt, H., Drusko, A., Renz, M. P., Schlömp, L., Tost, H., Schuh-Hofer, S., Tesarz, J., Meyer-Lindenberg, A., & Treede, R. D. (2025). Application of the grading system for "nociplastic pain" in chronic primary and chronic secondary pain conditions: a field study. *Pain*, *166*(1), 196–211. https://doi.org/10.1097/j.pain.0000000000003355

Tesarz, J., Herpel, C., Meischner, M., Drusko, A., Friederich, H. C., Flor, H., & Reichert, J. (2024). Effects of virtual reality on psychophysical measures of pain: superiority to imagination and nonimmersive conditions. *Pain*, *165*(4), 796–810. https://doi.org/10.1097/j.pain.0000000000003083
